# Integrin alpha-2 and beta-1 expression increases through multiple generations of the EDW01 patient-derived xenograft model of breast cancer—insight into their role in epithelial mesenchymal transition in vivo gained from an in vitro model system

**DOI:** 10.1186/s13058-020-01366-8

**Published:** 2020-12-04

**Authors:** Razan Wafai, Elizabeth D. Williams, Emma de Souza, Peter T. Simpson, Amy E. McCart Reed, Jamie R. Kutasovic, Mark Waltham, Cameron E. Snell, Tony Blick, Erik W. Thompson, Honor J. Hugo

**Affiliations:** 1 Invasion and Metastasis Unit, St. Vincent’s Institute, Melbourne, VIC Australia; 2 Department of Surgery, The University of Melbourne, St. Vincent’s Hospital, Melbourne, VIC Australia; 3 Queensland University of Technology, Institute of Health and Biomedical Innovation and School of Biomedical Sciences, Brisbane, QLD Australia; 4 Translational Research Institute, Brisbane, QLD Australia; 5 Australian Prostate Cancer Research Centre-Queensland and Queensland Bladder Cancer Initiative, Brisbane, QLD Australia; 6 The Royal Liverpool and Broadgreen University Hospitals NHS Trust, Liverpool, UK; 7 Centre for Clinical Research, Faculty of Medicine, University of Queensland, Brisbane, QLD Australia; 8 Monash University, Melbourne, VIC Australia; 9 Cancer Pathology Research Group, Mater Research Institute - The University of Queensland, Brisbane, QLD Australia; 10 Mater Pathology, Mater Hospital Brisbane, South Brisbane, QLD Australia

**Keywords:** Integrin, Hypoxia, Twist1, EMT, Breast cancer, Patient-derived xenograft (PDX)

## Abstract

**Background:**

Breast cancers acquire aggressive capabilities via epithelial to mesenchymal transition (EMT), in which various integrins/integrin-linked kinase signalling are upregulated.

**Methods:**

We investigated this in two patient-derived xenografts (PDXs) developed from breast-to-bone metastases, and its functional significance in a breast cancer cell line system. ED03 and EDW01 PDXs were grown subcutaneously in immunocompromised SCID mice through 11 passages and 7 passages, respectively. Tumour tissue was assessed using immunohistochemistry (IHC) for oestrogen receptor (ER)-alpha, E-cadherin, vimentin, Twist1, beta-catenin, P120-RasGAP, CD44, CD24 and Ki67, and RT-qPCR of EMT-related factors (*CDH1*, *VIM*, *CD44*, *CD24*), integrins beta 1 (*ITGB1*), alpha 2 (*ITGA2*) and *ILK*. Integrin and *ILK* expression in epidermal growth factor (EGF)-induced EMT of the PMC42-ET breast cancer cell line was assessed by RT-qPCR and Western blotting, as were the effects of their transient knockdown via small interfering RNA +/− EGF. Cell migration, changes in cell morphology and adhesion of siRNA-transfected PMC42-ET cells to various extracellular matrix (ECM) substrates was assessed.

**Results:**

The ED03 (ER+/PR−/HER2−/lobular) and EDW01 (ER+/PR−/HER2−/ductal) PDXs were both classified as molecular subtype luminal A. ED03 xenografts exhibited mutated E-cadherin with minimal expression, but remained vimentin-negative across all passages. In EDW01, the hypoxic indicator gene CAIX and Twist1 were co-ordinately upregulated at passages 4–5, corresponding with a decrease in E-cadherin. At passages 6–7, *VIM* was upregulated along with *ITGB1* and *ITGA2*, consistent with an increasing EMT. The ED03 PDX displayed minimal change over passages in mice, for all genes examined. *ILK*, *ITGB1* and *ITGA2* mRNAs were also increased in the EGF-induced EMT of PMC42-ET cells (in which *CDH1* was downregulated) although siRNA against these targets revealed that this induction was not necessary for the observed EMT. However, their knockdown significantly reduced EMT-associated adhesion and Transwell migration.

**Conclusion:**

Our data suggest that despite an increase in *ITGA2* and *ITGB1* gene expression in the EMT exhibited by EDW01 PDX over multiple generations, this pathway may not necessarily drive the EMT process.

**Supplementary information:**

The online version contains supplementary material available at 10.1186/s13058-020-01366-8.

## Introduction

Human breast cancer cell lines have been used extensively in vitro and in mice to dissect the cellular mechanisms associated with tumour aggressiveness and metastasis. However, cell line xenografts typically fail to recapitulate tumour cell heterogeneity. By contrast, human breast tumours engrafted into mice as patient-derived xenografts (PDXs) generally show excellent reproducibility of morphological and genetic characteristics of the original tumour with minimal genetic drift and as such are clinically relevant platforms for preclinical studies [1–5].

Aggressive cancers are known to display epithelial-mesenchymal plasticity (EMP), through which they can fluctuate between epithelial or mesenchymal states (or degrees of these) to assist survival in changing microenvironmental conditions [6–8]. Epithelial to mesenchymal transition (EMT) describes a phenotypic change toward a more mesenchymal state resulting in more motile and invasive cancer cells. EMT has been shown to promote progression in several cancer types including breast [9, 10], contributes to chemoresistance [11, 12], and is prominent in circulating tumour cells (CTCs) [7, 13–15]. Evidence for EMP has been demonstrated in numerous human breast cancer cell line studies and increasingly in breast cancer in vivo models [16–19] and clinical material (reviewed in [20]).

Invasive lobular carcinoma (ILC) and invasive ductal carcinoma (IDC) (also known as invasive carcinoma of no special type (IC NST)) have distinctive morphological features [21]. ILC is typified by single-file epithelial tumour cells, a finer stromal infiltration and often a minimal sclerotic tissue reaction—the combination of which makes self-detection and screen-detection (mammography) more difficult than for the typically more palpable IDC tumours, which grow as masses of epithelial cells within a desmoplastic stroma. These patterns are intimately linked to E-cadherin: ILCs do not express *CDH1* due to the presence of inactivating mutations [22, 23] or silencing via methylation [24], or copy loss [25], and hence grow as individual and linear arrays of tumour cells. By contrast, IDCs typically express *CDH1* and hence grow as cohesive tumour nests [21].

A major initiating event in the transcriptional programming of EMT is E-cadherin repression [26–28]. IDC expression of *CDH1* is downregulated when they undergo an EMT, which is associated with increased invasiveness [29, 30]; thus for the purposes of this study, IDC could be considered as “EMT-positive”. Interestingly, EMT does not occur spontaneously in ILC cells [31] and they tend to show less vimentin expression than IDC [32]; thus, ILC could be considered *“*EMT-negative”*.* Similarly, not all IDC-derived cell lines undergo EMT upon *CDH1* silencing, while some do [18].

Integrin switching is prominent in breast cancer EMT and has been linked to tumour aggressiveness [33], and integrins have been shown to play important roles in tumour cell transmigration via EMT (reviewed in [34]). Integrins are a large family of heterodimeric cell surface receptors that play a prominent role in the adhesive interactions between cells and their surrounding extra-cellular matrix (ECM), providing adhesion for stationary cells, as well as traction during cell movement [35–38]. TGFβ-induced EMT in NMuMG mouse mammary cancer cells results in the downregulation of epithelial *ITGA6*/*B4* expression (which mediates contact with the basement membrane) through epigenetically silencing of the gene encoding integrin β4 [39]. ITGA3/B1, which binds laminin and also associates with E-cadherin, is required for progression through EMT in lung alveolar epithelial cells, where it integrates beta-catenin and transforming growth factor-β (TGFβ)–SMAD signalling to promote myofibroblast formation and lung fibrosis [40]. Interactions between ITGA5/B1 integrin and fibronectin have been associated with EMT in EpH4 mouse normal mammary epithelial cells and human lung cancer cell lines [41, 42]. In pancreatic carcinoma cells, increased expression of ITGA1/B1 or ITGA2/B1 integrins and their interactions with type I collagen facilitate the disruption of E-cadherin complexes and the nuclear translocation of beta-catenin and promote proliferation and motility [43].

Indeed, ITGA2/B1 is widely expressed on epithelial cells and its levels are increased in several carcinoma cells from the epithelial origin [44]. Growing evidence indicates that ITGA2/B1 can be a key pathway in cancer pathogenesis [43, 45, 46]. Furthermore, Chen et al. showed that increased expression of ITGA2/B1 is positively correlated with increased metastatic ability in human squamous cell lung cancer cells when *i.v*. inoculated in severe combined immunodeficiency (SCID) mice [47].

It has been demonstrated that ILK can induce a complete EMT in various epithelial cell lines and thus be involved in the initiation of EMT in vivo, and the maintenance of the mesenchymal phenotype and disease progression [48–51]. ILK can modulate the expression of not only E-cadherin, but also other epithelial markers such as CK18 [52] and MUC1 [53], as well as mesenchymal markers such as LEF1 [54] and vimentin [53, 54]. Therefore, ILK is able to initiate an EMT. Gain and loss of function strategies have shown that over-expression, and/or constitutive activation of ILK results in oncogenic transformation and progression to invasive and metastatic phenotypes [55].

In the current study, the divergent cancer subtypes of ILC and IDC, which differ in regard to their EMT status, were studied as PDXs in order to examine the relationship between EMT, ITGA2/B1 and ILK in cancer progression, through serial passages in mice. The human tumoural material for both PDXs were obtained from bone metastases and thus may have already undergone cycles of EMT, and its reversal MET, to enable colonisation [26].

We coupled this with an investigation into the pattern of ILK and integrin expression changes in PMC42-ET human breast cancer cells induced by epidermal growth factor (EGF) treatment to undergo an EMT in vitro, and assessed whether these integrin changes were necessary for EMT to occur.

## Materials and methods

### Patient material and creation of xenografts

Establishment of the PDXs used in this study was described previously [56].

The ED03 xenograft was derived from a lobular breast cancer bone metastasis in a 40-year-old woman, 3.5 yrs. after initial diagnosis of her primary tumour. The EDW01 PDX was derived from a bone metastasis of an invasive ductal carcinoma of the breast presenting as clinically overt macrometastatic deposits in a 44-year-old woman.

Briefly, the tumour tissue derived initially from the bone metastasis deposits was diced into ~ 1 mm pieces, mixed with Matrigel® (BD Biosciences, Australia) and implanted bilaterally subcutaneously in SCID mice (ARC, Perth, Australia). For each passage, once tumour volumes reached 2000 mm^3^, mice were euthanised, and the tumours were removed. The tumour tissue was again chopped into chunks, mixed with Matrigel®, and implanted into fresh mice. This was repeated 6 times for EDW01 (total of 7 passages) and 10 times for ED03 (total of 11 passages). Portions were snap frozen for RNA extraction and formalin fixed and paraffin embedded for immunohistochemical analyses at each passage.

### Immunohistochemistry (IHC)

A tissue microarray was created of randomised duplicate 2-mm diameter cores of tumour blocks corresponding to various passage numbers through mice of the ED03 and EDW01 PDX model systems. IHC was performed using the Ventana Discovery Ultra Automated Slide Preparation system. Details of antibodies used in this study can be found in Table 1. The membrane-associated proteins (E-cadherin, beta-catenin, P120-RasGAP, CD24, CD44, carbonic anhydrase IX (CAIX)) were scored as cytoplasmic or membranous, and whether they were heterogeneous or homogeneous in these areas. Vimentin staining was scored as positive if present in the cytoplasm of cells, whereas for Twist1 and Ki67, the proportion of positive nuclei was recorded.

**Table 1:** Antibodies used in this study

	Antibody	Dilution	Supplier
**Antigen**			
Vimentin^^,#^	Mouse Monoclonal IgG (V9)	1:750	Dako, Australia
E-cadherin^^,#^	Mouse Monoclonal IgG (36)	1:12,500	BD Transduction Laboratories, USA
N-cadherin (A-CAM)	Mouse Monoclonal IgG (GC-4)	1:2000	Sigma-Aldrich, Australia
TWIST^^^	Mouse/ 2C1a	1:100	Abcam, England
Beta-catenin^^^	Mouse monoclonal, clone 14	1:500	BD Biosciences, Australia
P120^^^	Mouse monoclonal 98/pp120	1:200	BD Biosciences, Australia
CD24^^^	Mouse monoclonal SN3b	1:50	Thermo Fisher Scientific, Australia
CD44^^^	Mouse monoclonal 156-3C11	1:75	Abcam, England
Ki-67^^^	Mouse monoclonal MIB-1	1:100	Dako, Australia
CA-IX^^^	Rabbit monoclonal EP161	1:100	Cell Marque (Sigma Aldrich), USA
ITGB1^#^	Mouse Monoclonal IgG	1:5000	Chemicon International (Fisher Scientific), USA
ITGA2^#^	Rabbit Polyclonal	1:1000	Chemicon International (Fisher Scientific), USA
Integrin-Linked Kinase (ILK)^#^	Rabbit monoclonal IgG	1:4500	Cell Signaling Technologies (Danvers, MA,USA)
Pan-Actin, Ab-5^#^	Mouse Monoclonal IgG	1:10,000	Neomarkers (Invitrogen), USA
**Secondary antigen**			
Biotinylated Immunoglobulin^^,#^	Polyclonal Rabbit Anti-Mouse	1:200	Dako, Australia
IgG-HRP^^,#^	Goat Anti-Mouse	1:20,000	Dako, Australia
IgG-HRP^^,#^	Goat Anti-Rabbit	1:20,000	Dako, Australia

^^^Antibody used for IHC

^#^Antibody used for WB

Quantification of IHC data shown in Supplementary Figures 2 and 4 was determined using ImageJ, where DAB brown-positive nuclei were separated from blue (total) nuclei, and using the colour threshold tool, a threshold applied to only select positive nuclei that was visible by eye. Using this threshold, the area taken up by positive nuclei was quantified. The ratio of “relative intensity per cell” was obtained by dividing the overall area of positivity for the IHC target (either pink or brown) by overall nuclear area.

### Reverse transcriptase-quantitative PCR

RNA was extracted using the Qiagen RNeasy Mini prep Kit (Qiagen, Doncaster, VIC, Australia). cDNA synthesis and reverse transcriptase-quantitative PCR (RT-qPCR) were performed as previously described, using a specific reverse transcriptase (RT) primer in the cDNA synthesis step [57, 58]. Expression levels indicated by raw cycle thresholds (CTs) of the genes of interest were subtracted from the raw CT of the ribosomal protein L32 (*RPL32*) mRNA and plotted as dCT. *RPL32* CT values were observed to be unchanged by passage number in mice or PDX. Human-specific primers for various genes examined in this study are detailed in Table 2.

**Table 2:** QPCR primers for various genes examined in this study

Oligonucleotide name	Species	Sequence (5′-3′)
5′ Hs L32	Human	CAGGGTTCGTAGAAGATTCAAGGG
3′Hs L32	Human	CTTGGAGGAAACATTGTGAGCGATC
Hs L32 RT	Human	CAGAAAACGTGCACATGAGCTGC
5′ Hs CD24	Human	GACTCAGGCC AAGAAACGTC TTCTAAA
3′ Hs CD24	Human	GTTGCCTCTCCTTCATCTTG TACATGAAA
Hs CD24 RT	Human	GGGCGACAAAGTGAGACTGTCTAAAA
5′ Hs CD44	Human	CACAATGGCCCAGATGGAGAAA
3′ Hs CD44	Human	CTTCGACTGTTGACTGCAATGCAAA
Hs RT CD44	Human	GGCAATGTTGCAAGGGTTTGTGAAGACTT
5′ Hs VIM	Human	CAGGCGATATATTACCCAGGCAAGAA
3′ Hs VIM	Human	CTTGTAGGAGTGTCGGTTGTTAAGAA
Hs VIM RT	Human	CTAAATCTTGTAGGAGTGTCGGTTGTT
5′ Hs CDH1	Human	GGCACAGATGGTGTGATTACAGTCAAAA
3′ Hs CDH1	Human	GTCCCAGGCGTAGACCAAGAAA
Hs CDH1 RT	Human	CTCTGTCTTTGGCTGCAGCACTTTA
5′ Hs ILK	Human	GATGCAGGACAAGTAGGACTGGAA
3′ Hs ILK	Human	CAACCAGAGGCCTGCTGCTTT
Hs ILK RT	Human	GCTGGGGTAGTACCATGACTG
5′ Hs TWIST1	Human	CTAGAGACTCTGGAGCTGGATAACTAAAAA
3′ Hs TWIST1	Human	CGACCTCTTGAGAATGCATGCATGAAAAA
Hs TWIST1 RT	Human	GAGAAAGTCCATAGTGATGCCTTTCCTTT
5′ Hs SNAI1	Human	CCAGACCCACTCAGATGTCAAGAA
3′ Hs SNAI1	Human	GGCAGAGGACACAGAACCAGAAAA
Hs SNAI1 RT	Human	CGCAGACAGGCCAGCTCAGGAAT
5′ Hs SNAI2	Human	CCCAATGGCCTCTCTCCTCTTT
3′ Hs SNAI2	Human	CATCGCAGTGCAGCTGCTTATGTTT
Hs SNAI2 RT	Human	CATCGCAGTGCAGCTGCTTATGTTT
5′ Hs ZEB1	Human	GTTACCAGGGAGGAGCAGTGAAA
3′ Hs ZEB1	Human	GACAGCAGTGTCTTGTTGTTGTAGAAA
Hs ZEB1 RT	Human	GACAGCAGTGTCTTGTTGTTGTAGAAA
5′ Hs ZEB2	Human	CCACCTGGAACTCCAGATGCTTTT
3′ Hs ZEB2	Human	GCCTTGCCACACTCTGTGCATTT
Hs ZEB2 RT	Human	GCCTTGCCACACTCTGTGCATTT
5′ Hs ITGA2	Human	GACCTATCCACTGCCACATGTGAAAAA
3′ Hs ITGA2	Human	CCACAGAGGACCACATGTGAGAAAA
Hs ITGA2 RT	Human	GTCAGAACACACACCCGTTGTGTAATA
5′ Hs ITGB1	Human	GACTGATCAGTTCAGTTTGCTGTGTGTTT
3′ Hs ITGB1	Human	CCCTGCTTGTATACATTCTCCACATGATTT
Hs ITGB1 RT		CCCTGCTTGTATACATTCTCCACATGATTT
5′ Ms. ITGB1	Mouse	GCGTGTGCAGGTGTCGTGTTT
3′ Ms. ITGB1	Mouse	GAAGGCTCTGCACTGAACACATTCTTT
Ms ITGB1 RT	Mouse	GAAGGCTCTGCACTGAACACATTCTTT

### siRNA-mediated knockdown

Small interfering RNA (siRNA)-mediated knockdown of *ITGB1*, *ITGA2* and *ILK* (Horizon, [formerly Dharmacon], Melbourne, Australia) was performed in PMC42-ET cells. These cells display a molecular phenotype of Basal B (E Tomaskovic-Crook and T Blick, unpublished observation), based on clustering of a limited number of the Basal B discriminator genes [19] showing reliable data in an Affymetrix U133A analysis kindly performed by the laboratory of Joe Gray, Lawrence Berkeley National Laboratory, Berkeley, California [59]. These IDC-derived cells express E-cadherin mRNA and protein but do not assemble it at the cell membrane [27]. PMC42-ET were grown in RPMI with 10% foetal bovine serum (FBS, Thermo Fisher Scientific, Australia) at 37 °C with 5% CO_2_.

The siRNA target sequences for *ITGB1*, *ITGA2* and *ILK* are presented in Table 3. A commercial non-targeting control sequence (control siRNA) was also used (siSTABLE Non-targeting siRNA #1, Horizon, [formerly Dharmacon], Melbourne, Australia). Briefly, PMC42-ET cells were transfected using DharmaFECT4 (Horizon, [formerly Dharmacon], Melbourne, Australia) and 100 nM siRNA targeting ITGB1, ITGA2, ILK or control siRNA. The transfection efficiency of the siRNA was inferred by the level of protein knockdown that was achieved, determined by Western blot. Specificity of the siRNA knockdown of ITGB1, ITGA2 or ILK was ascertained by the lack of effects seen with a control siRNA, or against the other targets studied. After 8 h, cells were then left either unstimulated or stimulated with 10 ng/ml EGF for 72 h. Controls included cells alone (no transfection), transfection reagent alone and the control siRNA. Protein and RNA were extracted 72 h post EGF-stimulation and analysed by Western immunoblotting and RT-qPCR, respectively. Recombinant EGF was purchased from BD Biosciences, (Bedford, MA, USA).

**Table 3:** Sequences of siRNA used in the current study

siRNA constructs	siRNA sequence
ITGB1 siRNA	AAGCTTTTAATGATAATTCAT
ITGA2 siRNA	TCGCTAGTATTCCAACAGAAA
ILK siRNA	CCTGACGAAGCTCAACGAGAA

### Western blotting

Western blotting for ITGB1, ITGA2, ILK, pan-actin, Vimentin, N-cadherin and E-cadherin in siRNA transfected PMC42-ET cells +/− EGF was performed as previously described [57], with protein extracted using RIPA (radioimmunoprecipitation assay) buffer containing protease inhibitors. The RIPA-soluble fraction (supernatant after centrifugation) was analysed in all Western blots, with the insoluble pellet discarded. In regard to the contents of this RIPA buffer, 1.58 g Tris base and 1.8 g sodium chloride was dissolved in 150 ml of dH_2_0 and the pH adjusted to 7.4 with HCl. Twenty millilitres of 10% NP40 (Igepal) and 5 ml of 10% Na-deoxycholate (deoxycholic acid) was added and stirred until the mixture was clear. To this, 2 ml of 100 mM EDTA was added and the total volume adjusted to 200 mL with dH_2_0. One protease inhibitor cocktail tablet (Roche) was added to 10 mL RIPA immediately prior to use. Antibodies and their dilutions used for Western blotting are detailed in Table 1.

### Cell matrix adhesion assay

Wells of 24-well plates (polystyrene, non-tissue culture treated; Nunc Inc., Naperville, IL) were coated with 100 μg/ml collagen-I, 100 μg/ml collagen-IV, 20 μg/ml fibronectin or 50 μg/ml laminin. Collagen 1, collagen IV, laminin (from Engelbreth-Holm Swarm murine sarcoma) and fibronectin (from bovine plasma) used in these assays were all purchased from Sigma-Aldrich (St. Louis, MO, USA). Proteins were allowed to bind to the cells overnight at room temperature under the laminar flow hood, before the wells were rinsed with phosphate-buffered saline (PBS) and non-specific interactions were blocked for 1 h at 37 °C with 3% bovine serum albumin (BSA) in PBS, pH 7.4. PMC42-ET cells were transfected with siRNA. Eight hours later, cells were left either unstimulated or stimulated with EGF for 72 h, after which the cells were detached using 0.25% trypsin and allowed to attach to the various (ECM substrate-coated plates for 1 h. Cells attached to ECM was estimated by the average of cell counts from five random high-power fields under light microscopy (counted in situ on the substrate after washing).

### Monolayer wound healing assay

This was performed as previously described [57]. Briefly, PMC42-ET cells were plated in a 6-well plate set up in triplicate and incubated at 37 °C for 24 h to allow the formation of a confluent monolayer. The cells were then wounded by using a P200 pipette tip. The wounded monolayers were washed with complete media to remove detached cells. Images of the wounds were taken at 0, 24 and 48 h. Wound areas at each time point were analysed and quantitated using ImageJ software.

### Boyden chamber migration assay

Boyden chamber migration assays were performed as previously described [60]. Briefly, transmigration culture assays were performed using 8-μm pore Transwell chambers (Corning, USA). Polycarbonate membranes (8-μm pore size) of the upper compartment of 24-well chambers were coated with 100 μg/ml collagen I in serum-free media (SFM; Roswell Park Memorial Institute [RPMI]-1640 medium). siRNA-transduced PMC42-ET cells (+/− EGF) harvested by trypsinisation were re-suspended in SFM supplemented with 0.2% BSA, and the cell suspension (2.5 × 10^5^ cells suspended in 250 μl SFM) was applied to the upper compartment in triplicate wells. The lower compartment was filled with 650 μl of chemoattractant (RPMI-1640 containing 10% FBS [Sigma-Aldrich]). After 24 h of incubation, the chambers were rinsed in PBS to eliminate non-adherent cells and the remaining non-migrated cells on the upper surface of the filter were removed carefully with a cotton swab. Migrated cells on the lower side of the filter were stained with 0.5% crystal violet (Sigma Aldrich, Australia) for 15 min. The crystal violet dye retained on the filters after washing was extracted with 10% acetic acid and cell migration was measured by reading the absorbance at 560 nm on a micro-titre plate reader (PolarStar Optima, BMG Labtech, Ortenberg, Germany).

For use of the ILK inhibitor QLT0267 in Boyden Chambers, sub-confluent monolayers of the PMC42-ET cells were left either untreated or treated for 24 h with this inhibitor (QLT, Inc., Vancouver, Canada) at a final concentration of 6.25 μM prior to assay. Invasion of PMC42-ET cells in vitro was assessed by the invasion of the cells through Collagen-I-coated Transwell inserts. The inhibitor was dissolved in DMSO (0.1%), which was used as the vehicle control.

### Statistical analyses

Gene expression data across PDX passages were analysed using the two-tailed Mann-Whitney test (non-parametric) and Pearson’s correlation co-efficient, and the IHC intensity changes across PDX passages were analysed using Ordinary One-Way ANOVA. Cell matrix adhesion and Boyden chamber migration assay results were analysed using two-way ANOVA with Dunnett’s multiple comparison test. All statistical analyses were performed using GraphPad Prism v7 (GraphPad Software, San Diego, USA).

## Results

### Histological comparison of the ED03 and EDW01 xenografts with increasing passages through mice

**Fig. 1:**
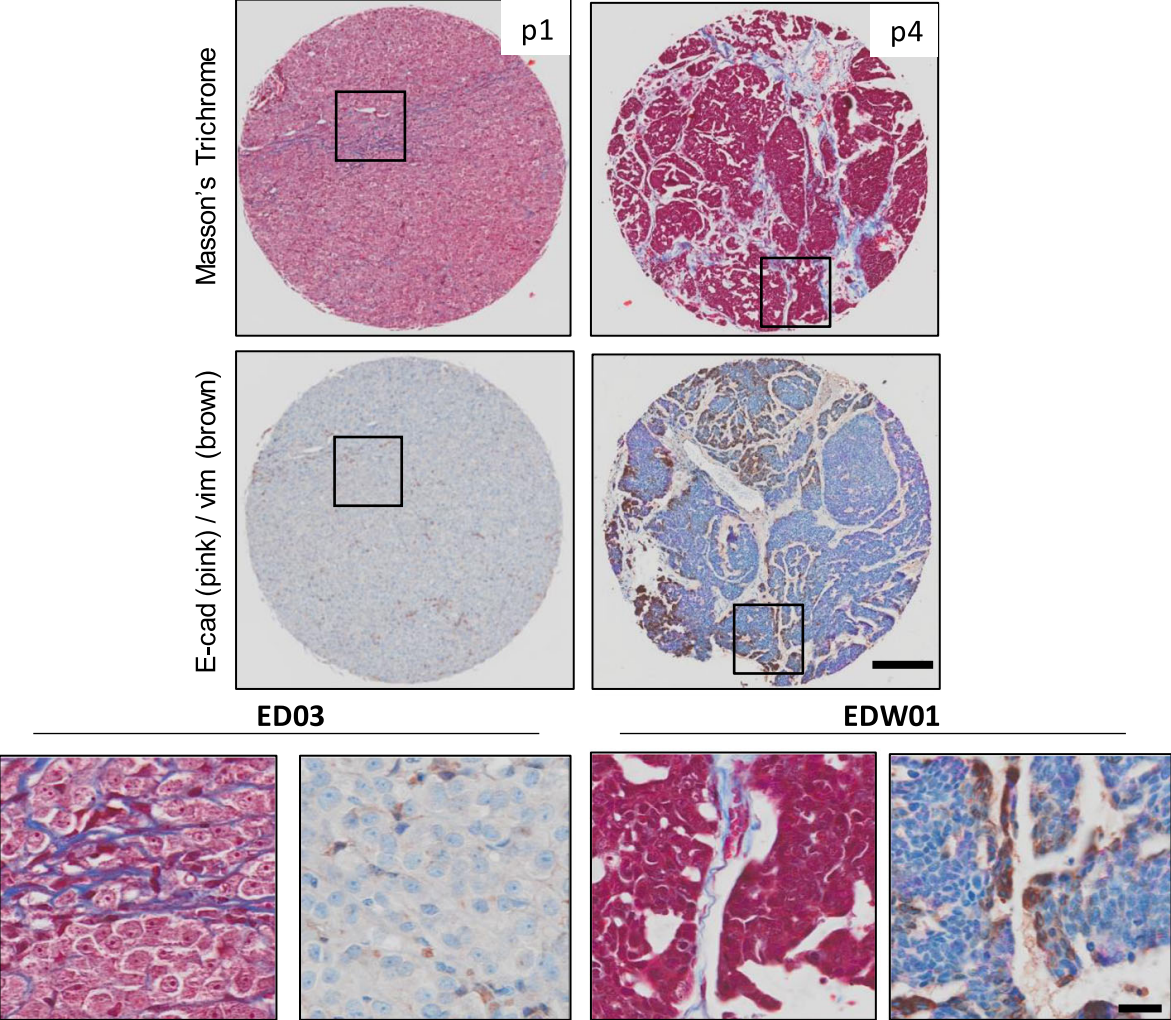
Masson’s trichrome and E-cadherin (E-cad)/vimentin (vim) immunohistochemistry performed on serial section from ED03 (LHS; passage 1 (p1)), and EDW01 (RHS; passage 4 (p4)) PDX models. Boxed areas shown as higher magnification images displayed below images of whole cores. Scale bar upper panels, 200 μm; lower panels 20 μm. H&E stained sections from donor blocks from which cores were derived can be found in Supplementary Fig. 2

The ED03 PDXs was serially passaged up to passage (p) 11, whereas the EDW01 PDX was passaged up to p7. Histologically, the ED03 xenografts displayed a diffuse growth pattern with minimal visible tumour stroma, often growing in long cords of cells, consistent of ILC. By contrast, EDW01 PDX revealed histology consistent with IDC, with clearly visible stromal septae separating growing tumour islands (Fig. 1). In ED03, the stromal collagen was evident only under higher magnification as it was finer and more pericellular compared with EDW01, in which the thicker stromal cords separated lobules of tumour. This stroma was of murine origin, as it did not stain with human-specific vimentin antibody (Fig. 1). Abundance of stromal area observed for EDW01 shown in here in Fig. 1 did not visibly increase with subsequent passaging in mice (Supplementary Fig. 1). Tumoural cores examined were generally representative of histology observed in the donor blocks from which the tissue microarray was assembled (Supplementary Fig. 2).

### Assessment of oestrogen receptor (ERα) across serial passages

**Fig. 2:**
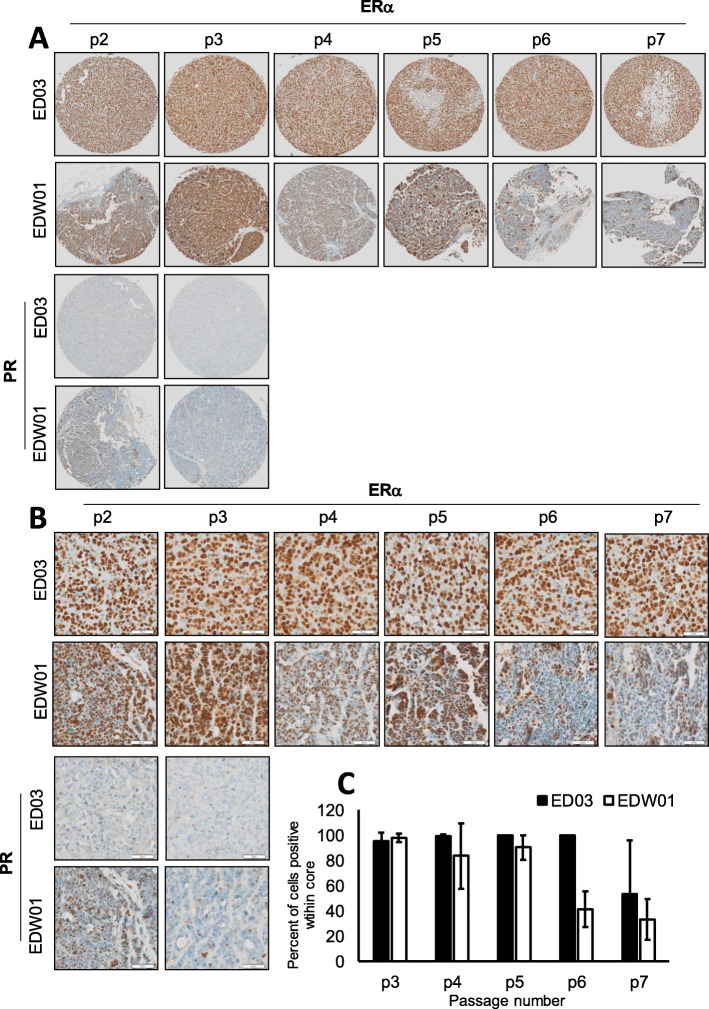
Immunohistochemical analysis (brown) of ED03 and EDW01 PDXs at various passages in mice for oestrogen receptor alpha (ERα) and progesterone receptor (PR). Nuclei counterstained with haematoxylin. **a** 4× magnification, scale bar, 200 μm. **b** 10× magnification, scale bar, 50 μM. **c** Plot of average % positivity per core for each PDX per passage number. ED03: p3; *n* = 2, p4; *n* = 4, p5; *n* = 4, p6; *n =* 2, p7; n = 4. EDW01: p3; *n* = 8, p4; *n* = 10, p4: *n* = 8, p5; *n* = 7, p6; *n* = 3, p7; *n* = 3. H&E stained sections from a subset of donor blocks from which cores were derived can be found in Supplementary Fig. 2

**Fig. 3:**
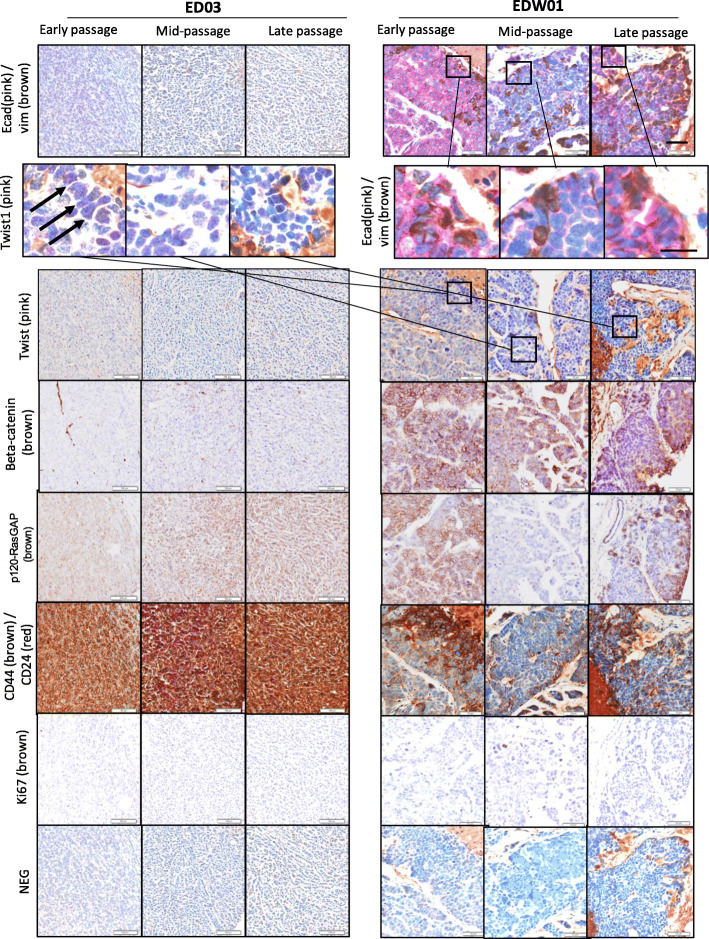
Epithelial and mesenchymal marker analysis of the ED03 & EDW01 PDX tumours over serial passages (ED03 early: passage 3, mid: passage 7, late: passage 11; EDW01 early: passage 3, mid: passage 5, late: passage 7). Nuclei counterstained with haematoxylin. NEG: negative control (concentration and isotype matched IgG substituted for primary antibody) Arrows indicate Twist1-positive nuclei. 10× magnification, scale bar, 50 μm. Row 2 selected higher magnification areas: scale bar, 20 μm. H&E stained sections from a subset of donor blocks from which cores were derived can be found in Supplementary Fig. 2

We investigated the expression of ERα in the ED03 and EDW01 PDX models. As shown in Fig. 2 (low power in part A, higher power shown in part B), immuno-reactivity to ERα in the ED03 PDX was approximately 99% tumour cells, and this level of staining was maintained until p7, where ERα was found to be low in some of the TMA cores. The EDW01 PDX also displayed almost 100% positivity for ERα at p3; however, this progressively declined to approximately 40% in passages 6 and 7. These relative changes are plotted in Fig. 2c. Progesterone receptor (PR) expression was negative in ED03 in all passages and was weak (< 15%) in EDW01 at p3, disappearing by p4. HER-2 in both PDXs was negative (data not shown). The clinical approximated subtypes of breast cancer (defined according to the 2011 St Gallen International Breast Cancer Conference) classify both ED03 and EDW01 as Luminal A, since Ki67 is less than 14% in both PDXs (Fig. 3) [61].

### Immunohistochemistry (IHC) and RT-qPCR quantification of EMT markers

**Fig. 4:**
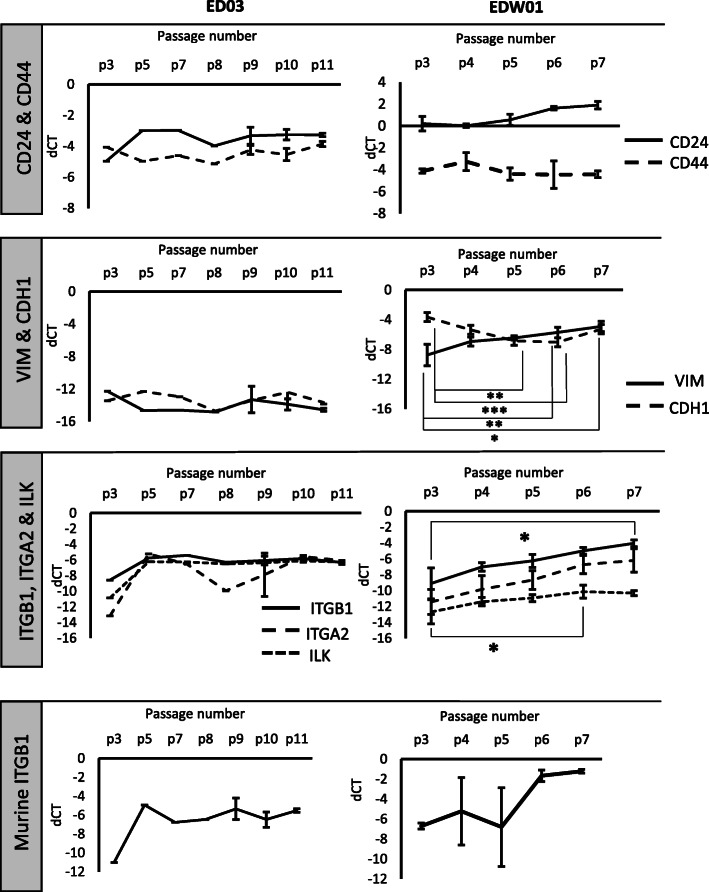
Gene expression (RT-qPCR) for the ED03 and EDW01 PDX models. Mean of individual xenograft tumours are shown, where *n* ≥ 3 error bars are shown (standard error of the mean). For ED03: p3 *n* = 1, p5 *n* = 1, p7 *n* = 2, p8 *n* = 1, p9 *n* = 3, p10 *n* = 3, *p* = 11 *n* = 3. For EDW01: *VIM/CDH1* and for *ITGAB1/A2/ILK*: p3 *n* = 6, p4 *n* = 11, p5 *n* = 6, p6 *n* = 11, p7 *n* = 3; for *CD24/CD44* and murine *Itgb1*, all passages were *n* = 3. dCT in the figure represents delta CT, or change in CT values calculated by subtracting the raw CT of the gene of interest from the raw CT of the housekeeping gene *RPL32*. Statistical significance was calculated using two-tailed Mann-Whitney test (Non-parametric). *p < 0.05, **p < 0.01, ***p < 0.001

To assess any changes in EMT status over sequential passaging, key effector molecules implicated in the EMT process (vimentin/E-cadherin, Twist1, beta-catenin, P120-RasGTPase activating protein [P120-RasGAP], CD24/CD44) and the proliferative marker Ki67 were screened in the ED03 and EDW01 PDX models across the series of passages using IHC (Fig. 3) and human-specific RT-qPCR (Fig. 4).

E-cadherin immunostaining was almost completely absent in ED03 original patient material, consistent with its lobular carcinoma derivation [23]. We subsequently confirmed a putative somatic missense variant (p.His128Asn, *data not shown*). Consistent with this, less than 1% of cells expressed E-cadherin in any ED03 PDX passage in mice (Fig. 3, top left panel). This was reflected in a relative E-cadherin staining intensity index per cell score of the range 0–0.2 compared with 0.2–0.9 for EDW01 (Supplementary Fig. 3). Similarly, beta-catenin was not detectable within the ED03 PDXs; p120-RasGAP was aberrant, with staining observed to be mostly cytoplasmic. By contrast, EDW01 PDXs displayed strong E-cadherin immunostaining (Fig. 3, top right panel) and readily detectable RNA levels (Fig. 4). However, this was accompanied by a progressive increase in human-specific *VIM* mRNA expression with each passage (Fig. 4): passage 6 material displayed a significant increase (*p* = 0.008), and passage 7 material also displayed significant increase (*p* = 0.024) in comparison to passage 3 material. This was consistent with a significant (*p* > 0.05) increase in vimentin protein intensity per cell index across the passages for EDW01, derived from IHC (Supplementary Fig. 3). E-cadherin-positive tumour cells transitioning to vimentin positivity (possibly remaining E-cadherin positive, see high magnification inset, Fig. 3) were observed in EDW01 PDXs whereas *VIM* mRNA and protein in ED03 were almost negligible (Figs. 3 and 4 Supplementary Fig. 3).

As shown in Fig. 3, positive nuclear Twist1 expression was seen only in EDW01 xenograft tumours, adjacent to regions of necrosis (as indicated by black arrows); however, nuclear Twist1 positivity did not increase in abundance across the passages (Supplementary Fig. 3). Beta-catenin and P120-RasGAP was also mainly observed in EDW01 at the cell membrane and corresponded with E-cadherin staining.

We went on to further examine the expression of breast cancer stem cell markers *CD44* and *CD24*, as upregulation of *CD44* and downregulation of *CD24* is observed in breast cancer cell line EMT [19]. ED03 displayed a homogeneous CD44 IHC pattern, which was relatively consistent throughout the passages at the protein and mRNA level (Figs. 3 and 4, Supplementary Fig. 4–5). By contrast, CD44 protein was heterogeneously expressed in EDW01. EDW01 exhibited higher mRNA abundance overall than ED03 (Fig. 4), consistent with the appearance (Fig. 3) and quantification of protein abundance by IHC where the increase in CD24 was found to be significant with increasing passage number (Supplementary Fig. 5). Within EDW01, but not ED03, there were regions of tumour cells that appeared to lack both CD24 and CD44 (Fig. 3); however, these regions in EDW01 that are negative for CD44/CD24 do not increase over passage number (Supplementary Fig. 4).

A parallel study of integrin expression in the PMC42-ET breast cancer cell line induced to undergo EMT with EGF indicated that *ITGA2* and *ITGB1*, and their downstream regulator *ILK*, appeared to be upregulated (Supplementary Fig. 6)*.* Hence *ILK* and these integrins were examined further in the PDX models*.* Increases in *ITGA2* (p7 significantly higher than p3) and *ITGB1* (p6 significantly higher than p3) were observed in ED03 xenograft material, which were maintained (Fig. 4). However, similar to the increased vimentin seen with each passage in EDW01, the levels of human *ITGB1* mRNA in the xenografts increased with successive passage, demonstrating significantly higher expression (*p* = 0.026) at passage 6 in comparison to passage 3 (Fig. 4). Furthermore, *ITGA2* mRNA expression in EDW01 xenografts (Fig. 4) was significantly upregulated at passage 7 material when compared to passage 3 (*p* = 0.024). ILK is activated by integrins including ΙΤΓΑ2/Β1 and mediates a number of signalling responses in relation to survival and proliferation in addition to induction of EMT [62]. A trend was observed toward upregulation of *ILK* mRNA expression in both ED03 and EDW01 xenografts (Fig. 4). Murine (stromal) *Itgb1* displayed a similar pattern of upregulation as human (tumoural) *ITGβ1* (Fig. 4).

These findings suggest that with serial passage EDW01 has accrued features consistent with EMT. The co-induction of the mRNA levels of *ITGB1* and *ITGA2* in EDW01 indicates that they may be important for the EMT process and/or phenotype, because they track with the indices of EMT (decreased E-cadherin and increased vimentin) observed in this model system.

### Further investigation of EMT drivers and markers in the EDW01 xenograft model

Hypoxia is a common driver of EMT in breast cancer, and E-cadherin repressor genes have been implicated in this process [63]. We sought to examine the pattern of *SNAI1*, *SNAI2*, *TWIST1* and *ZEB1/2* expression through the serial passages in mice in the ED03 and EDW01 xenograft models, in comparison to the hypoxic indicator gene carbonic anhydrase 9 (*CAIX*).

**Fig. 5:**
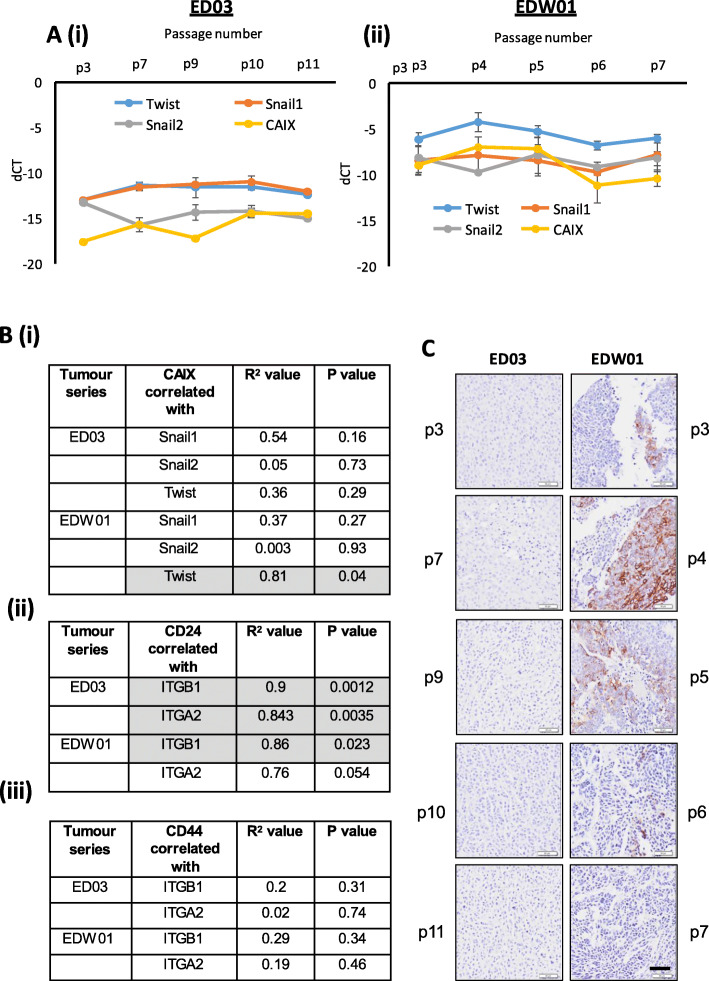
**a** Quantitative, real-time RT-PCR gene expression of various E-cadherin transcriptional repressor genes *(TWIST1*, *SNAI1*, *SNAI2)* and correlation with expression of the hypoxic indicator gene Carbonic Anhydrase 9 *(CAIX)* in RNA extracted from tumours in the ED03 and EDW01 PDX models across serial passages. Mean expression values shown, error bars are standard error from the mean; mRNA from 3 xenografts per passage was analysed. dCT in the figure represents delta CT, or change in CT values calculated by subtracting the raw CT of the gene of interest from the raw CT of the housekeeping gene *RPL32*. **b** (i) Pearson correlation statistics of data shown in **a**. Pearson’s correlation statistics of *ITGB1* and *ITGA2* correlated with (ii) *CD24* or (iii) *CD44* in the PDX models. **c** Carbonic Anhydrase 9 immunohistochemisty in the ED03 and EDW01 PDX models with passage (p) numbers shown in the vertical plane. Images shown are representative of the following numbers of xenografts within their respective PDX passage numbers: ED03—p3: 6; p7: 7; p9: 1; p10: 1, p11: 1; EDW01—p3: 8; p4: 13; p5: 8; p6: 7; p7: 3. *p* value shown is two-tailed, and *p* < 0.05 defined as statistically significant and shaded grey in **b**. Scale bar, 50 μm

As shown in Fig. 5, of the E-cadherin repressor genes examined (*SNAI1*, *SNAI2*, *TWIST1* and *ZEB1/2*), *TWIST1* was more highly expressed in the EDW01 xenograft compared with ED03 (Fig. 5a, (i)). *ZEB1* and *ZEB2* were not expressed at detectable levels in either PDX. Both *TWIST1* and *CAIX* appeared to exhibit a similar expression pattern across the passages in the EDW01 PDX (Fig. 5a, (ii)). Pearson correlation analyses of this data (shown in Fig. 5b, (i)) indicated that this relationship was significant (*R*^2^ = 0.81, *p* = 0.04). Furthermore, the pattern of membrane intensity of CAIX (Fig. 5c), where we observed CAIX to be increase at p4 then drop at p6 in the EDW01 PDX, appeared to align with the *CAIX* (and *TWIST1*) gene expression data (shown in Fig. 5a, (ii)). This suggests that Twist1 may be somewhat functionally involved in the hypoxia-induced EMT through consecutive passages of the EDW01 PDX; however, further investigation is needed.

**Fig. 6:**
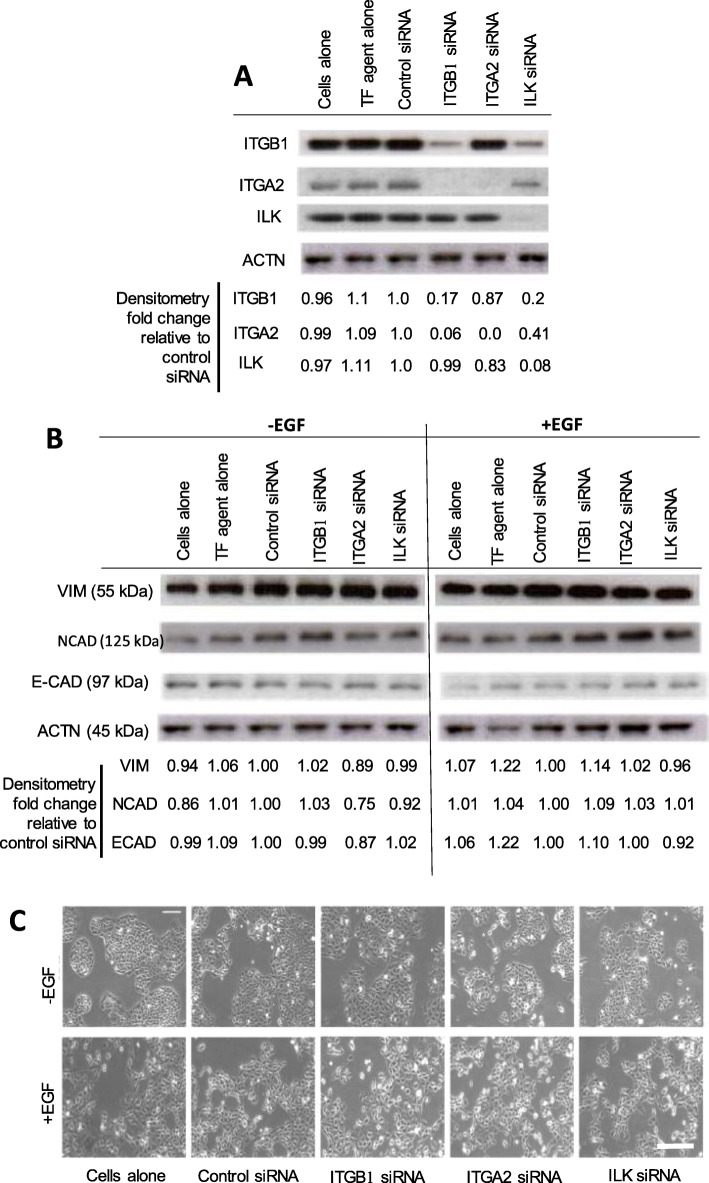
**a** Western blots showing ITGB1 (130 kDa), ITGA2 (160 kDa) and ILK (50 kDa) versus pan-actin (ACTN, 45 kDa) protein expression of siRNA transfected PMC42-ET cells; siRNA knockdown did not affect EMT of PMC42-ET cells in response to 10 ng/ml EGF treatment (+EGF) after 72 h as shown by **b**. western blotting for vimentin, (53 kDa), NCAD (100 kDa) and E-cadherin (120 kDa); numbers shown below the Western blots in **a** and **b** represent respective protein expression after normalisation to pan actin, relative to cells treated with control siRNA; TF: Transfection. **c** Phase contrast cellular morphology of cells from which protein was extracted for Western blots shown in **b**. Scale bar, 100 μm. The results of experiments shown are representative of three independent experiments

CD24 is an epithelial-associated marker with relevance to breast cancer stem cells, where its expression is reduced in comparison to luminal breast cancer cells [19]; its expression has been shown to indirectly stimulate cell adhesion to fibronectin, collagens I and IV and laminin through the activation of integrin activity [64]. Interestingly, the expression pattern of *CD24* with *ITGB1* was significantly positively correlated in the ED03 series (*R*^2^ = 0.9, *p* = 0.0012) and in the EDW01 series (*R*^2^ = 0.96, *p* = 0.023). *CD24* was also positively correlated with *ITGA2* in the ED03 series (*R*^2^ = 0.84, *p* = 0.0035) and this reached near significance in the EDW01 series (*R*^2^ = 0.76, *p* = 0.054) (Fig. 6c). No significant or near significant correlations were observed for *CD44* with integrins in either PDX systems (Fig. 5b, (iii)).

### Functional assessment of candidate genes ITGB1, ITGA2 and ILK in the PMC42-ET system

As expression of the *ITGA2/B1* components were associated with the EMT observed in EDW01 xenografts over serial passages through mice, we tested whether they could be a “driver” of the EMT, using the PMC42-ET EMT model system.

Although already somewhat mesenchymal [27], PMC42-ET cells treated with EGF in vitro undergo a further EMT in which *ILK*, *ITGB1* and *ITGA2* are upregulated (supplementary Fig. 4), [63]. We examined the effects of siRNA knockdown of *ITGA2*, *ITGB1* and *ILK* on late-stage mesenchymal gene expression, cell adhesion and cell migration.

As shown in Fig. 6a, individual siRNA knockdown of *ITGB1*, *ITGA2* or *ILK* resulted in the expected reduction of expression of the target genes. In addition, *ILK* inhibition also led to the reduction of ITGB1 protein levels by 80% (0.2 in Fig. 6a); inhibition of *ITGB1* also led to reduced ITGA2 protein expression (0.06 in Fig. 6a); and *ILK* inhibition led to a reduction in ITGA2 protein expression by 60% (0.41 in Fig. 6a). Inhibition of *ITGB1* and *ITGA2* by siRNA did not affect ILK protein levels. These data indicate a complex interplay between these three components.

We observed a greater dynamic range in the induction of vimentin with EGF after 72-h treatment in Supplementary Fig. 6C compared to Fig. 6B. This may be ascribed to differing behaviours of cells in culture over time and according to confluency when passaging but also differing gel/film exposure times between the two experiments. Despite the observable difference in regard to vimentin, one can deduce a clear and somewhat comparable downregulation of E-cadherin in both experiments.

To determine whether the cells with suppressed *ITGB1*, *ITGA2* or *ILK* were able to undergo EMT with EGF treatments, cellular morphology and protein expression was examined. After 72 h of EGF treatment, cellular morphology (Fig. 6c) revealed a clear acquisition of spindle-shapes and breaking apart of cellular islands, consistent with an EMT. Protein expression of the “classical” indicators of EMT, vimentin, E-cadherin and N-cadherin were measured by Western immunoblotting (Fig. 6b). E-cadherin was dramatically reduced in the “cells alone” treated with EGF compared to the untreated “cells alone” control, whereas slight increases in vimentin and N-cadherin were observed in these untransfected cells, consistent with EGF-induced EMT of these cells as previously reported [27, 63].

No observable differences in the responses of vimentin, N-cadherin or E-cadherin protein levels to 72 h of EGF treatment were seen following treatment with *ITGB1*, *ITGA2* or *ILK* siRNA, when compared to treatment with control siRNA (Fig. 6b). Similarly, the mesenchymal morphology caused by EGF treatment was not abrogated by any of the siRNAs (Fig. 6c).

Together, it seems to suggest that these candidates do not directly mediate the EMT induced by EGF in this breast cancer cell line.

We then investigated what effect the stepwise increase in integrin expression, observed in the EDW01 xenografts (Fig. 4), may have had on growth and invasion/motility of these tumours over serial passages in mice, inferred by parallel analyses in the PMC42-ET cells. We focused on cell adhesion to various substrates and migration, properties known to be mediated by integrins and ILK, and these were again examined in PMC42-ET cells.

**Fig. 7:**
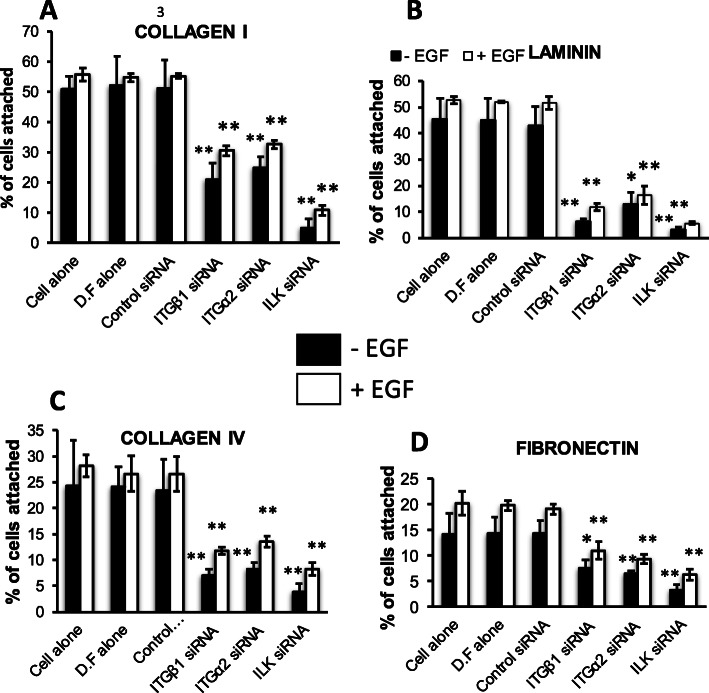
Inhibition of PMC42-ET breast cancer cell adhesion to **a** collagen I, **b** collagen IV, **c** laminin and **d** fibronectin, by integrin subunit-specific and ILK siRNAs. Results are expressed as % of cells adhered and represent the mean ± SD from 4 biological replicates. Results were analysed using two-way ANOVA, with Dunnett’s multiple comparison test, with *P* values adjusted for multiple comparisons; *p < 0.01, **p < 0.001, ***p < 0.0001

PMC42-ET cells require ITGB1, ITGA2 and ILK for maximal adherence to collagen I, collagen IV, laminin and fibronectin substrates, as knockdown of these molecules significantly reduced adhesion in comparison to control siRNA (Fig. 7a–d, *p* < 0.05). When stimulated with EGF, the attachment of the PMC42-ET cells treated with *ITGB1*, *ITGA2* and *ILK* siRNA was also significantly abrogated (*p* < 0.001).

**Fig. 8:**
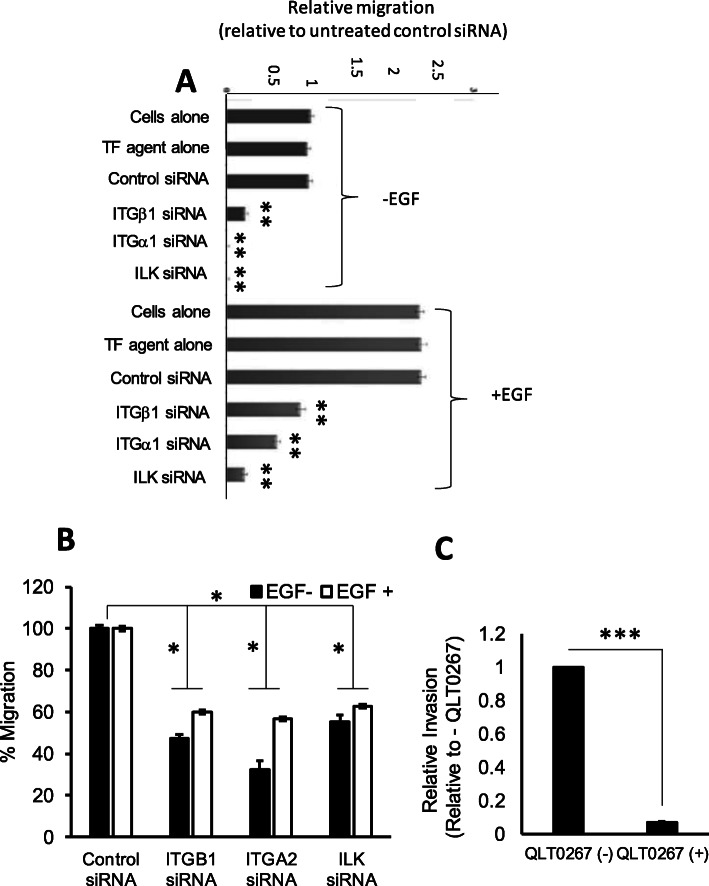
Migration properties of PMC42-ET cells following siRNA knockdown of *ITGB1*, *ITGA2* and *ILK* without and with stimulation by EGF. Effect on migration was assessed using the **a** Boyden Chamber and **b** scratch-wound assays. Data are expressed as percentage of control and represent the mean ± SD from 3 biological replicates. **c** Effect of ILK inhibitor QLT0267 (used at 6.25 μM, in DMSO) on PMC42-ET cell migration in the Boyden Chamber assay. Results represent average cell counts from of 5 random high-power fields from three independent experiments, error bars represent standard deviation. For **a**, statistical significance (*p < 0.05, comparison to control siRNA) was determined using two-way ANOVA, with Dunnett’s multiple comparison test. For **b**, statistical significance was determined using two-way ANOVA followed by Holm-Sidak’s multiple comparisons test. For **c**, statistical significance (***p < 0.0001) was determined using unpaired two-tailed *t* test with Welch’s correction

Coordinated regulation of cell adhesion and adhesion complex remodelling are crucial for cell movement. *ITGB1*, *ITGA2* and *ILK* siRNA-mediated silencing in PMC42-ET cells caused them to be significantly less migratory, as shown by the assessment of migration using the Boyden Chamber Assay (Fig. 8a) and the Monolayer Wound Healing assay (Fig. 8b). Inhibition of ILK by a specific inhibitor, QLT0267, also significantly reduced cellular movement in the Boyden Chamber Assay (Fig. 8c, *p* < 0.001). EGF treatment caused these cells to increase their migration, whereas *ITGB1*, *ITGA2* and *ILK* silencing each significantly reduced migration in both assays under EGF-stimulated conditions (Fig. 8a, b, *p* < 0.05).

### Evaluation of ITGB1, ITGA2 and ILK with respect to clinical parameters in breast cancers: analysis of a published dataset

**Fig. 9:**
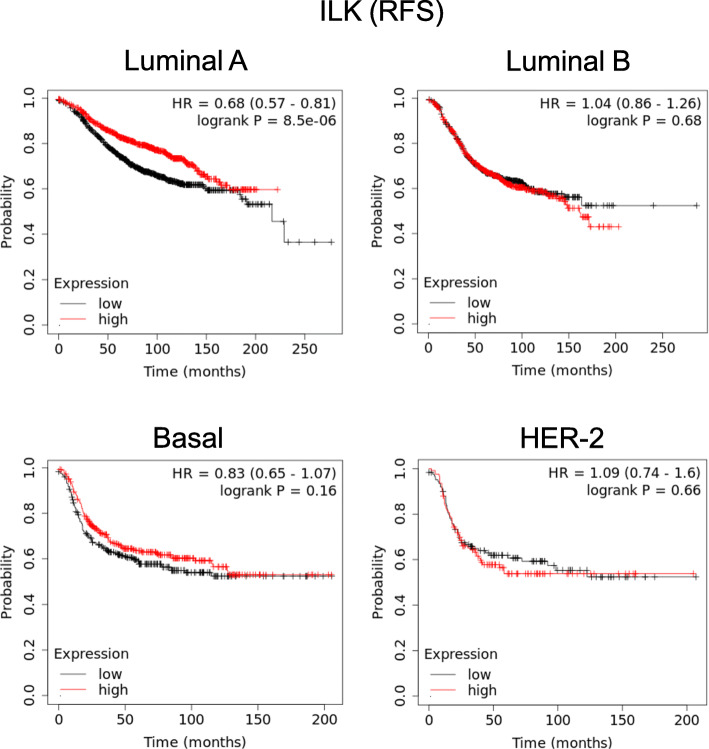
Kaplan-Meier curves of regression-free survival data examining expression of ILK across various breast cancers of varying molecular phenotypes. *P* values of ITGB1 and ITGA2 along with ILK to predict other clinical parameters in Luminal A tumours are shown in Supplementary Fig. 7. Data acquired from a publically available database [65]

Gene expression levels of ITGA2, ITGB1 and ILK were assessed across a previously published breast cancer cohort [65] with respect to regression-free survival (RFS), overall survival (OS), distant metastasis-free survival (DMFS) and progression-free survival (PFS), specifically examining Luminal A breast cancers. Of the genes examined, only ILK was found to be significant, and only for regression-free survival, with high expression being predictive of this clinical parameter. These results are shown in tabular form in Supplementary Fig. 7. Kaplan-Meier curves for ILK in regard to RFS are shown in Fig. 9. Its expression in Luminal A breast cancers is shown compared to RFS in other breast cancer molecular subtypes of Luminal B, Basal and Her-2. The results show that ILK specifically predicts improved RFS in Luminal A breast cancers.

## Discussion

We have shown that the EDW01 PDX model displayed evidence of EMT with progressive passages through mice, which was not seen in ED03. This is consistent with the known EMT status of IDC versus ILC, of which EDW01 and ED03 are examples, respectively. However, a comparison of the progression of such divergent cancer types with regard to EMT status has enabled the discovery of some unique findings. The partial EMT observed in EDW01 was associated with a rising hypoxia leading to Twist1 expression in early-mid passages, repressing E-cadherin expression and orchestrating vimentin upregulation, and accompanied by upregulation of *ITGB1* and *ITGA2* expression. The mesenchymal shift appeared to then return to the epithelial direction in later passages of EDW01; however, the increased integrin expression persisted. We present cell line data to support the association of ITGA2/B1 and the ILK signalling pathway with the observed EMT, but the EMT was not mediated by these. Assessment of ITGA2/B1 and ILK expression in a published Luminal breast cancer dataset was not consistent with the pro-aggressive implications of our association with EMT in the EDW-01 PDX, since ILK predicted improved regression-free survival post treatment. Thus, further analysis of this association in other models is required to refine the interpretation.

Although the EMT-associated ITGB1, ITGA2 and ILK are not essential for EGF-induced EMT in PMC42-ET cells (Fig. 6), they are necessary for breast cancer cell adhesion to ECM-substrates (Fig. 7) and cellular movement (Fig. 8). These molecules were more significantly upregulated in increasing EDW01 passages through mice than ED03 (Figs. 3 and 4); thus, they may have enabled ECM adhesion in this PDX. Indeed, previous studies demonstrate that ITGA2/B1 is primarily a receptor for collagen and laminin [66] and expression is also associated with motility, invasiveness, and cellular differentiation of a variety of tumours [67, 68]. This is in contrast to studies in which ITGA2 and ITGB1 have been found to suppress metastasis in models of mouse and human cancer [69]. However, Dedhar and Saulnier [70] showed that the expression of *ITGA2/B1* increased in the chemically transformed human osteosarcoma cells, and this integrin was implicated in tumour progression and metastasis. Similarly, *ITGA2/B1* expression accelerated either experimental metastasis or tumour dissemination of melanoma [71] and rhabdomyosarcoma [72, 73], gastric cancer [74, 75] and colon cancer cells [76]. Taken together, our data suggest that ITGA2/B1 contribute to the EMT phenotype observed increasingly in EDW01 over serial passages in mice.

The PDX models of ED03 and EDW01 were characterised as luminal A (ER positive, Her2 negative, PR low or absent) whereas the PMC42-ET breast cancer cell line used in this study was of the Basal B molecular phenotype [63]. However, luminal A cell lines lack the degree of plasticity that is clearly evident in the EDW-01 PDX, so we felt it was justified to compare the PMC42-ET cell line that also exhibits plasticity. The PMC42-ET cells also largely exhibit an epithelioid appearance, despite constitutive vimentin-expression, and we have shown that they respond to EMT-inducing regimens such as EGF. We acknowledge that this is a limitation of our study which directly influences the conclusions that we can logically make in regard to the importance of ITGA2/B1 in cancer progression.

Actively growing tumours acquire areas of hypoxia, a product of imperfect angiogenesis coupled with rapid growth, which can facilitate cellular invasion via the induction of EMT [77]. Vimentin and Twist1 positivity was observed in close proximity to necrotic areas in early passages and less commonly found at the centre of tumour ‘islands’ (Fig. 3). Of the E-cadherin repressor genes examined, *TWIST1*, a target of HIF1A [78–80] displayed the strongest correlative pattern of induction with *CAIX* (Fig. 6b, *R*^2^ = 0.81, *p* = 0.04). Induction of *TWIST1* coincided with the repression of *CDH1* and induction of *VIM* mRNA and therefore may be the instigator of the observed EMT in the EDW01 xenograft model. Indeed, hypoxia has been implicated in inducing EMT-related genes in another PDX model of serial transplantation. Wegner and colleagues [81] demonstrate in their cervical cancer PDX model serially transplanted in mice that the EMT orchestrating gene *SNAI1* and stem cell markers were found to be increased in late compared to early passages along with hypoxic *CAIX* gene expression, accompanied with an increase in tumour aggressiveness and proliferative rate. Their finding, in a different cancer type (cervical), adds further support to our suggestion that hypoxia may have been a major driving force in the observed progressive EMT in the EDW01 PDX model.

However, why did the mesenchymal shift return to epithelial in later passages of EDW01? Tumours in vivo have been found to adapt to low oxygen environments, such as reprogramming Akt signalling in the mitochondria [82]. This coupled with the well-known ability of tumours to increase angiogenesis [83] contributes to tumour cell survival and progression. Although beyond the scope of this investigation, the EDW01 PDX model provides a means to investigate these phenomena further, with relevance to understanding the progression of breast cancer in women.

Although many studies have associated EMT with therapy resistance [11, 84], it is important to note that the EDW01 xenograft was not challenged by therapy; the EMT progression was spontaneous. Interestingly, considerable emphasis is being placed recently on the hybrid state of EMP recently [6, 85–88], and this phenotype appears to manifest in the EDW01 xenografts (elevated vimentin and apparently only partially lost E-cadherin as shown in Figs. 3 and 4). A separate analysis of circulating tumour cells (CTCs) in the ED03 model indicate that despite the lack of any evidence of EMT in the primary xenograft tumours, the CTCs are enriched in mesenchymal gene expression, but also in epithelial genes (*CDH1* and *CD24*), compared to the primary tumour, indicating a dysregulation of this axis and/or possibility of hybrid cells [58].

Tumour-stroma crosstalk plays an integral role in EMT in vivo [89]; similarly, we observed key changes in murine stroma in the PDX models examined in this study. We have previously demonstrated that EDW01 evoked greater expression of *MMPs* (*-2*, *-9*, *-11* and *MT1-MMP*) in the murine stroma than ED03 [56]. Furthermore, EDW01 displayed MT1-MMP and MMP-13 at the tumour-stromal boundary, but did not express these factors or MMP-2 and MMP-9 within the tumour mass itself. As shown in the current study, the EDW01 tumours grew as islands traversed by thick collagenous stromal bands whereas the ED03 had delicate pericellular stroma dispersed throughout (Fig. 1). This pattern of growth may be directly attributable to the pattern of MMP expression of each of these PDX models—EDW01 lacked the capacity to invade as individual cells, possibly due to the lack of induction of intratumoural MMP-2 and MMP-9. Furthermore, the murine microenvironment (non-orthotopic) in which the EMT occurred in the EDW01 PDX over successive passages may have been conducive to this change. We found that murine (stromal) *Itgb1* expression aligned with human (tumoural) expression of the same integrin; in fact for EDW01, stromal *Itgb1* expression was approximately 22-fold higher than tumoural *ITGB1* at passage 7 (Fig. 4). This leads to speculation as to whether the murine microenvironment was the instigator of the EMT or a responder in this process. However, given that an EMT was not observed in the ED03 line, which was passaged through mice of the same genotype (SCID), it could be postulated that drivers of EMT came from within the tumour itself, lending further support to the notion that hypoxia was an initiating event.

Decreased CD44/CD24 expression ratio in later passages in both PDX lines was an unexpected finding, at least in EDW01, as CD24^+/high^/CD44^low−^ phenotype is associated with the epithelial phenotype despite EMT being observed in this xenograft model. However, CD24 expression can also confer adhesive properties enabling invasion. In a meta-analysis of 16 studies of 5697 breast cancers, CD24 was found to be significantly associated with poorer survival [90], presumably due to non EMP functions. In studies on breast cancer cell lines in vivo, CD24 was found to act as a ligand for P-selectin on the lung vascular endothelium [64]. We found that *CD24*, but not *CD44*, correlated with *ITGA2* and *ITGB1* in both PDX models (Fig. 5b, ii versus iii), providing further suggestion, in addition to our PMC42-ET integrin knockdown/EGF studies (Fig. 6), that activation of these integrins is not necessarily intimately related to the EMT process. We recognise the limitation that the PMC42-ET cellular behaviour was examined in two-dimensional culture, and as such, our observations are hypothesis generating and require further validation.

Posttranslational cleavage of CD44 may explain the discrepancy between CD44 protein expression between the two PDXs—EDW01 *CD44* gene expression is comparable with ED03 (Fig. 4) whereas CD44 membranous protein expression is strong and uniform in ED03 but somewhat weaker and more heterogeneous in EDW01 (Fig. 3, Supplementary Fig. 4–5). CD44 may be shed from the cell by the action of MMPs, namely MMP-9 and MT1-MMP [91, 92], resulting in the loss of cell membrane CD44. The CD44 antibody used in this study (clone 156-3C11, Abcam) recognises cell-membrane localised CD44. As previously mentioned in our earlier studies, EDW01 evoked greater expression of MMPs (-2, -9, -11 and MT1-MMP) in the murine stroma than ED03 [56]. As shown in Supplementary Fig. 4, the homogeneous versus heterogeneous CD44 expression in ED03 compared with EDW01 associates with the interruption of growing tumour by murine stroma, as illustrated by Masson’s Trichrome staining, where connective tissue stains blue. A greater level of CD44 cleavage and shedding may have occurred in EDW01 PDX tumours than ED03, facilitated by stromal MMPs, resulting in the observed heterogeneous pattern. MMP-directed CD44 cleavage results in nuclear translocation of the intracellular CD44 domain [93], which can result in the transcriptional activation of EMT-associated genes [94, 95] and induction of stemness [96]. Nuclear CD44 has been shown to occupy the *TWIST1* promoter [96]; therefore, CD44 cleavage in EDW01 could have contributed to *TWIST1* transcriptional upregulation (Fig. 5a).

## Conclusion

This study illustrates a cumulative EMT in a PDX system through several passages in mice, an effect characterised by, but not orchestrated by, ILK signalling via ITGA2 and ITGB1, and possibly ILK.

## Supplementary information

**Additional file 1:** **Supplementary Fig. 1.** Stromal area in EDW01 tumour cores across serial passaging in mice does not increase. Connective tissue visualised by the Masson’s Trichrome stain, × 4 magnification, scale bar = 200 μM.

**Additional file 2:** **Supplementary Fig. 2.** H&E stained sections of the donor blocks from which representative duplicate cores were taken for creation of a tissue micro-array recipient block, which was then used for all immunohistochemistry and histochemical staining depicted in this manuscript. Part A depicts donor blocks used for cores shown in Fig. 1, part B depicts donor blocks used for cores shown in Fig. 2A, and part C depicts donor blocks used for cores shown in Fig. 3. 0.4x magnification, scale bar = 2 mm. 0.8x magnification, scale bar = 1 mm.

**Additional file 3:** **Supplementary Fig. 3.** Image J-based quantification of IHC targets that displayed the greatest visual change between PDXs and across passages 3 to 7 for both PDXs: E-cadherin, Vimentin and Twist1. Relative intensity per cell was calculated by dividing the overall area of DAB positivity for the IHC target by overall nuclear area. Statistical significance was determined by an Ordinary one-way ANOVA, where * indicates *p* < 0.05 and ** indicates *p* < 0.005.

**Additional file 4:** **Supplementary Fig. 4.** A Representative images of CD44 and Masson’s Trichrome for the the ED03 and EDW01 PDXs at the indicated passage numbers. The numbers of xenografts examined at various passage numbers are as follows: for ED03 – p3: 6; p5: 5; p7: 7; for EDW01 – p3: 8; p5: 8; p7: 3. Magnification 4x, scale bar = 200 μM. B. CD44/24 images shown in Fig. 3, split into their respective colours to illustrate staining. Magnification 10x, scale bar = 100 μM.

**Additional file 5:** **Supplementary Fig. 5.** Image J-based quantification of relative intensity per cell of IHC targets that displayed the greatest visual change between PDXs and across passages 3 to 7 for both PDXs: CD24 and CD44. Relative intensity per cell was calculated by dividing the overall area of DAB positivity for the IHC target by overall nuclear area. Statistical significance was determined by an Ordinary one-way ANOVA, where * indicates p < 0.05 and ** indicates p < 0.005.

**Additional file 6:** **Supplementary Fig. 6** EGF treatment (10 ng/ml, 72 h) of PMC42-ET breast cancer cells resulted in an EMT which associated with an upregulation (trend only) of ITGB1, ITGA2 and ILK. A. Phase contrast morphology, B. gene expression changes as assessed by RT-qPCR. Results are from one experiment, representative of two independent experiments. Error bars are standard deviation of *n* = 3 technical replicates within 1 biological replicate (1 experiment). C. Western blotting for vimentin, E-cadherin, and actin across the EGF time course. Scale bar, 100 μm.

**Additional file 7:** **Supplementary Fig. 7.** Assessment of clinical parameters (Regression Free Survival-RFS, Overall Survival-OS, Distant Metastasis Free Survival-DMFS and Progression Free Survival-PFS) in Luminal A cancers with respect to high versus low ITGB1, ITGA2 or ILK expression, as derived from a previously published database [65].

## Data Availability

All data generated or analysed during this study are included in this published article and its supplementary information files.
