# Supplementary figures and images for: Integrin alpha-2 and beta-1 expression increases through multiple generations of the EDW01 patient-derived xenograft model of breast cancer—insight into their role in epithelial mesenchymal transition in vivo gained from an in vitro model system
Source: Breast Cancer Res. 2020 Dec 4;22:136. doi: 10.1186/s13058-020-01366-8 (PMC7716465; doi:10.1186/s13058-020-01366-8)

**EDW01**

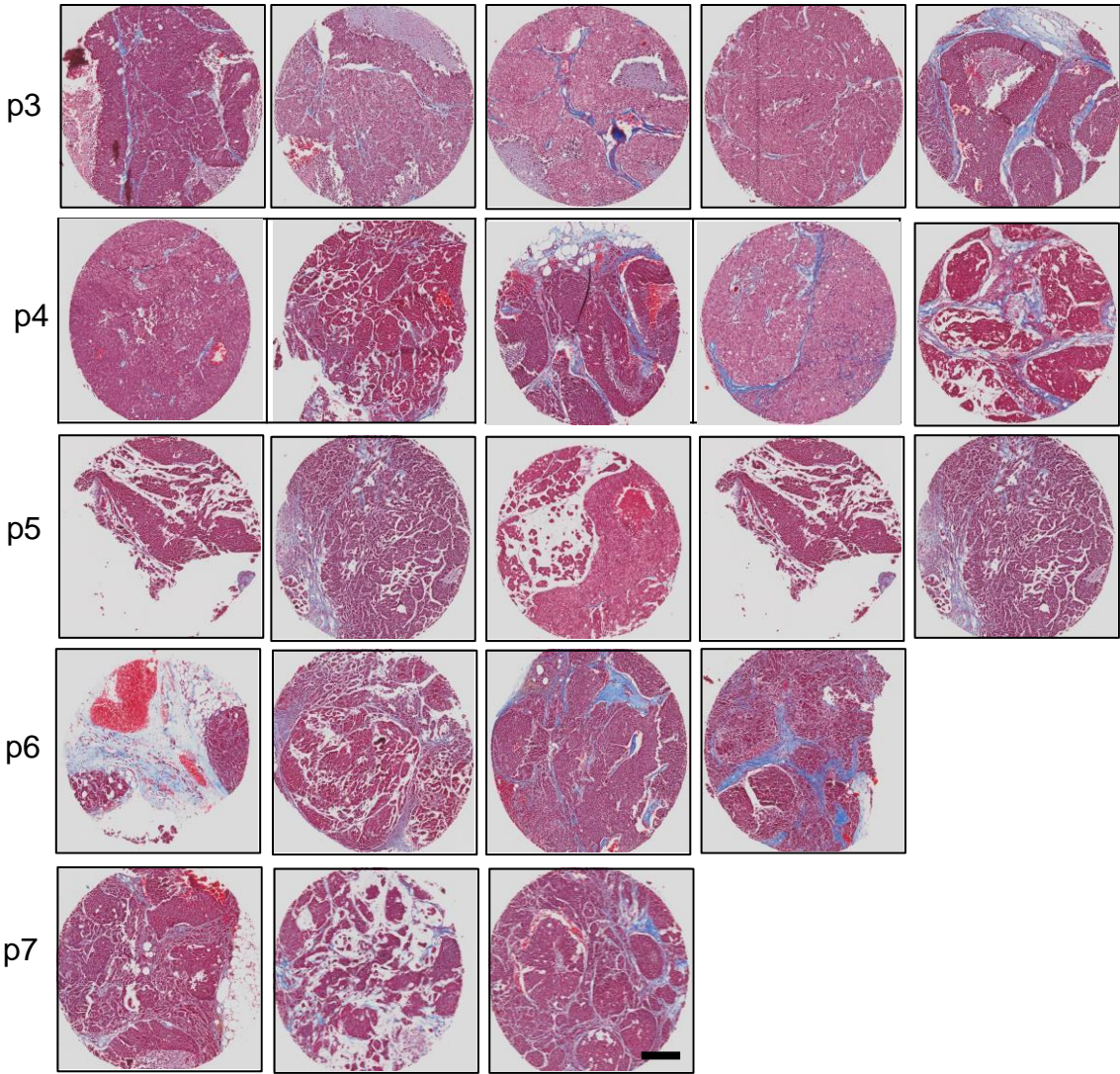

Supplement: Supplementary file 1 — Additional file 1: Supplementary Fig. 1. Stromal area in EDW01 tumour cores across serial passaging in mice does not increase. Connective tissue visualised by the Masson’s Trichrome stain, × 4 magnification, scale bar = 200 μM. [file 13058_2020_1366_MOESM1_ESM.pdf]

Donor blocks for Figure 1

ED03

0.4x

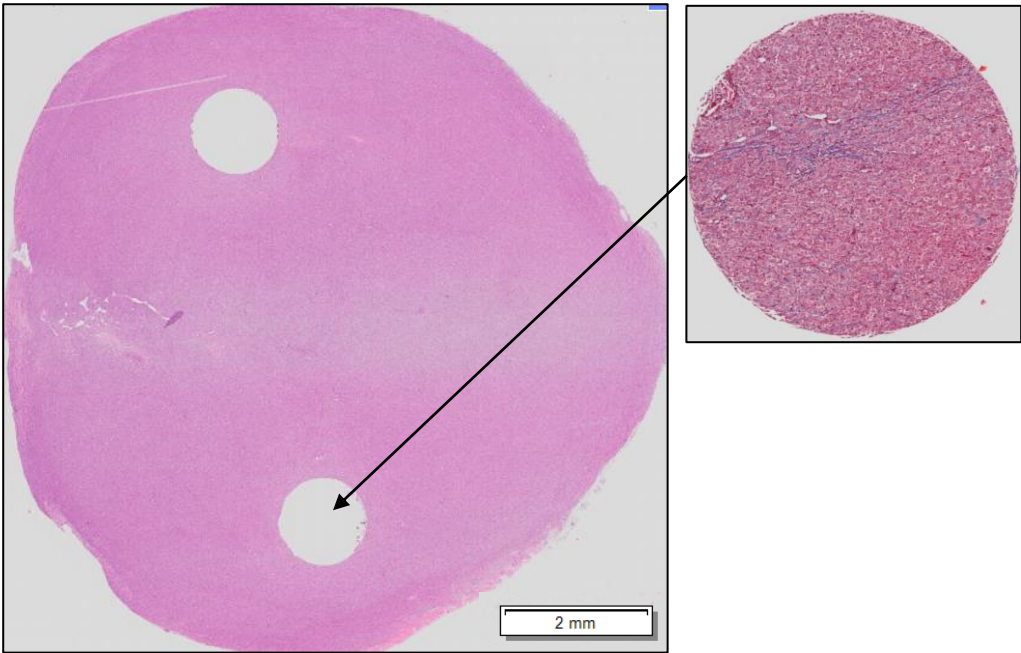

EDW01

0.8x

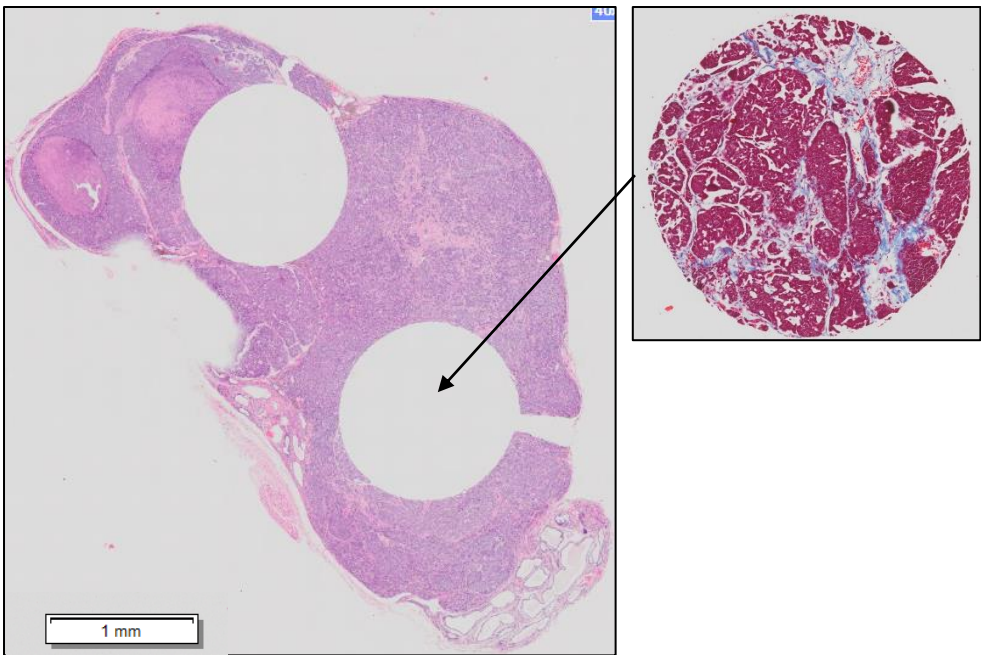

Supplement: Supplementary file 2 — Additional file 2: Supplementary Fig. 2. H&E stained sections of the donor blocks from which representative duplicate cores were taken for creation of a tissue micro-array recipient block, which was then used for all immunohistochemistry and histochemical staining depicted in this manuscript. Part A depicts donor blocks used for cores shown in Fig. 1, part B depicts donor blocks used for cores shown in Fig. 2A, and part C depicts donor blocks used for cores shown in Fig. 3. 0.4x magnification, scale bar = 2 mm. 0.8x magnification, scale bar = 1 mm. [file 13058_2020_1366_MOESM2_ESM.zip › Supplementary Figure 2A.pdf]

Donor blocks for Figure 3

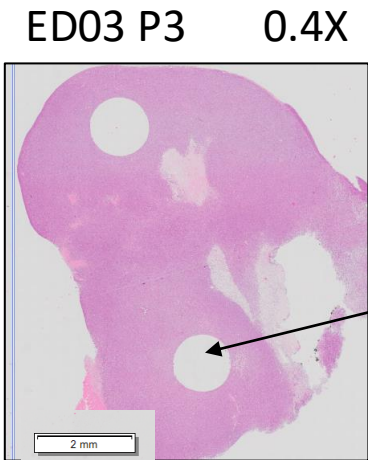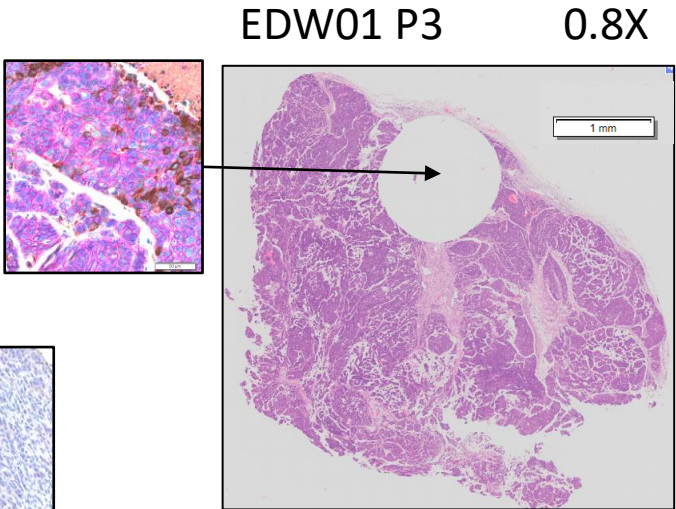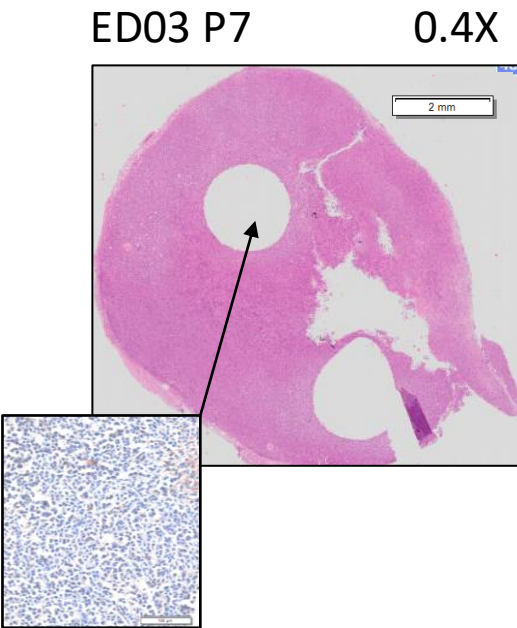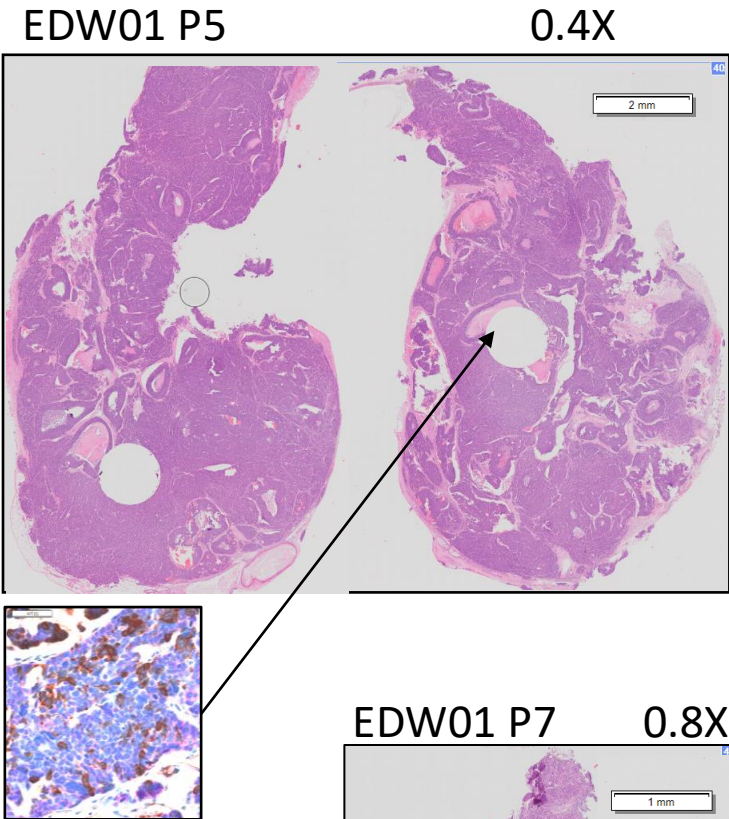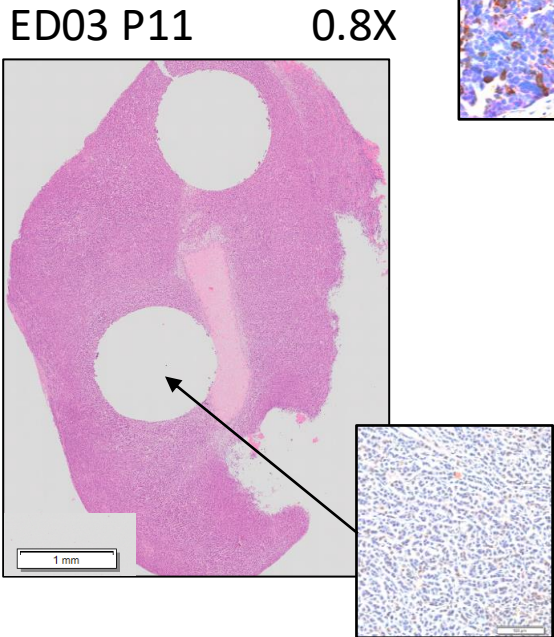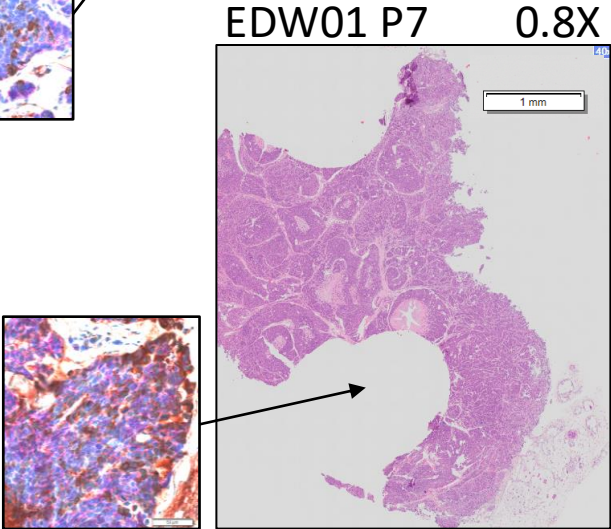

Supplement: Supplementary file 2 — Additional file 2: Supplementary Fig. 2. H&E stained sections of the donor blocks from which representative duplicate cores were taken for creation of a tissue micro-array recipient block, which was then used for all immunohistochemistry and histochemical staining depicted in this manuscript. Part A depicts donor blocks used for cores shown in Fig. 1, part B depicts donor blocks used for cores shown in Fig. 2A, and part C depicts donor blocks used for cores shown in Fig. 3. 0.4x magnification, scale bar = 2 mm. 0.8x magnification, scale bar = 1 mm. [file 13058_2020_1366_MOESM2_ESM.zip › Supplementary Figure 2C.pdf]

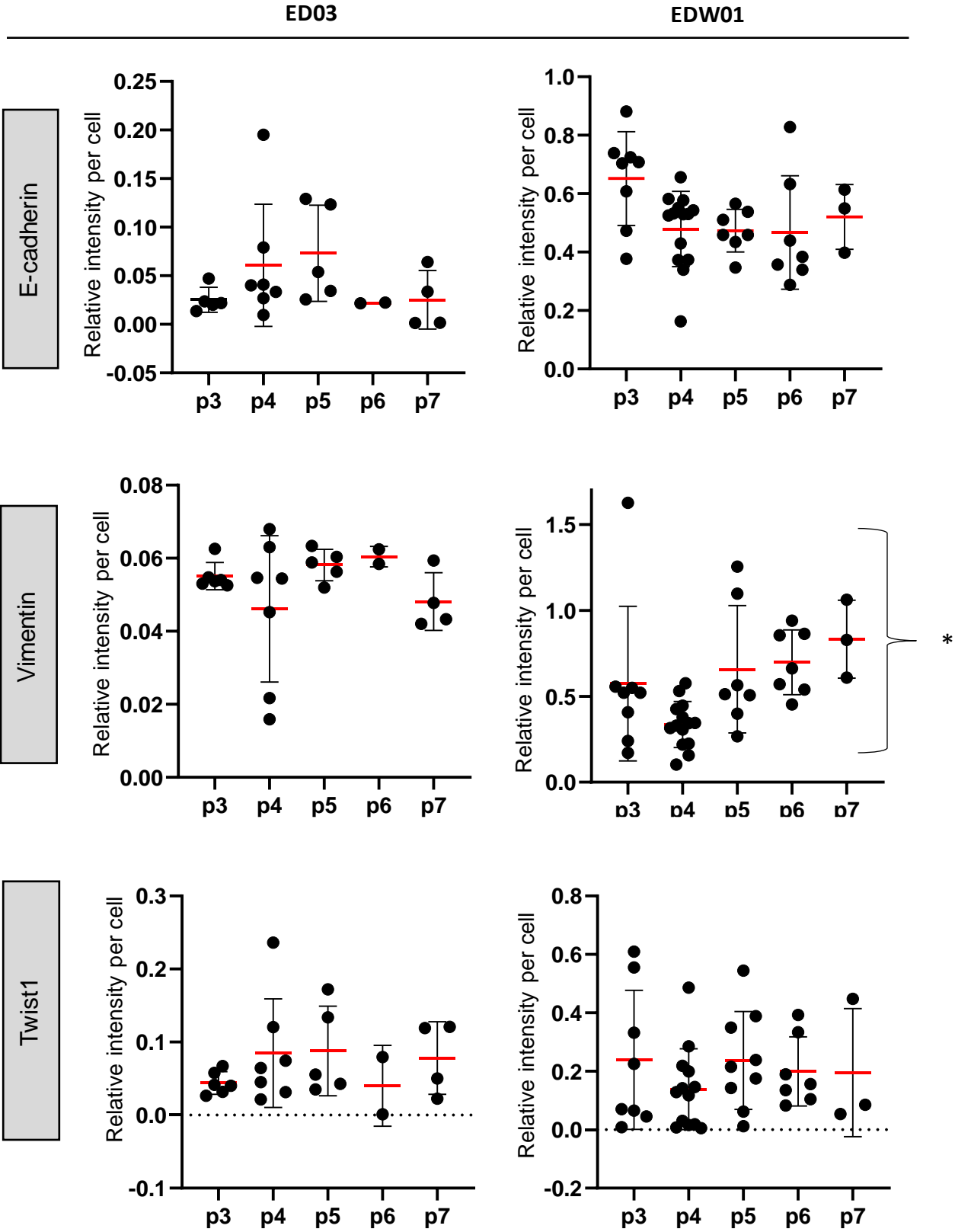

Supplement: Supplementary file 3 — Additional file 3: Supplementary Fig. 3. Image J-based quantification of IHC targets that displayed the greatest visual change between PDXs and across passages 3 to 7 for both PDXs: E-cadherin, Vimentin and Twist1. Relative intensity per cell was calculated by dividing the overall area of DAB positivity for the IHC target by overall nuclear area. Statistical significance was determined by an Ordinary one-way ANOVA, where * indicates p < 0.05 and ** indicates p < 0.005. [file 13058_2020_1366_MOESM3_ESM.pdf]

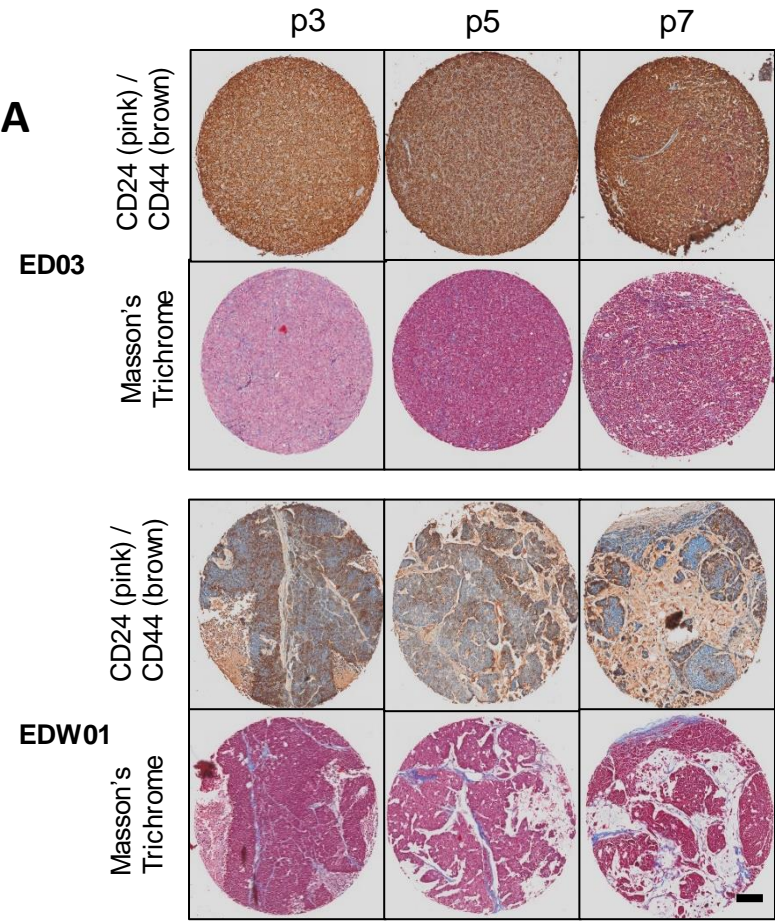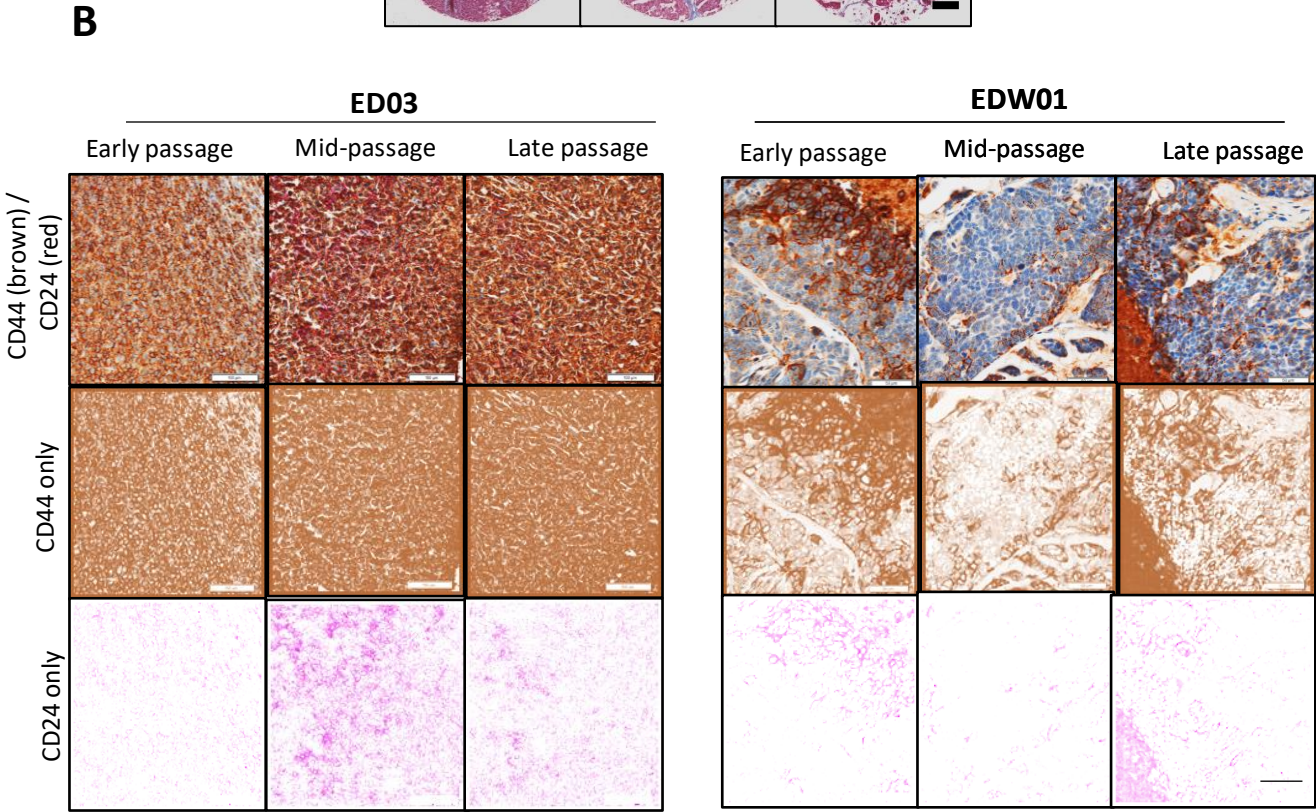

Supplement: Supplementary file 4 — Additional file 4: Supplementary Fig. 4. A Representative images of CD44 and Masson’s Trichrome for the the ED03 and EDW01 PDXs at the indicated passage numbers. The numbers of xenografts examined at various passage numbers are as follows: for ED03 – p3: 6; p5: 5; p7: 7; for EDW01 – p3: 8; p5: 8; p7: 3. Magnification 4x, scale bar = 200 μM. B. CD44/24 images shown in Fig. 3, split into their respective colours to illustrate staining. Magnification 10x, scale bar = 100 μM. [file 13058_2020_1366_MOESM4_ESM.pdf]

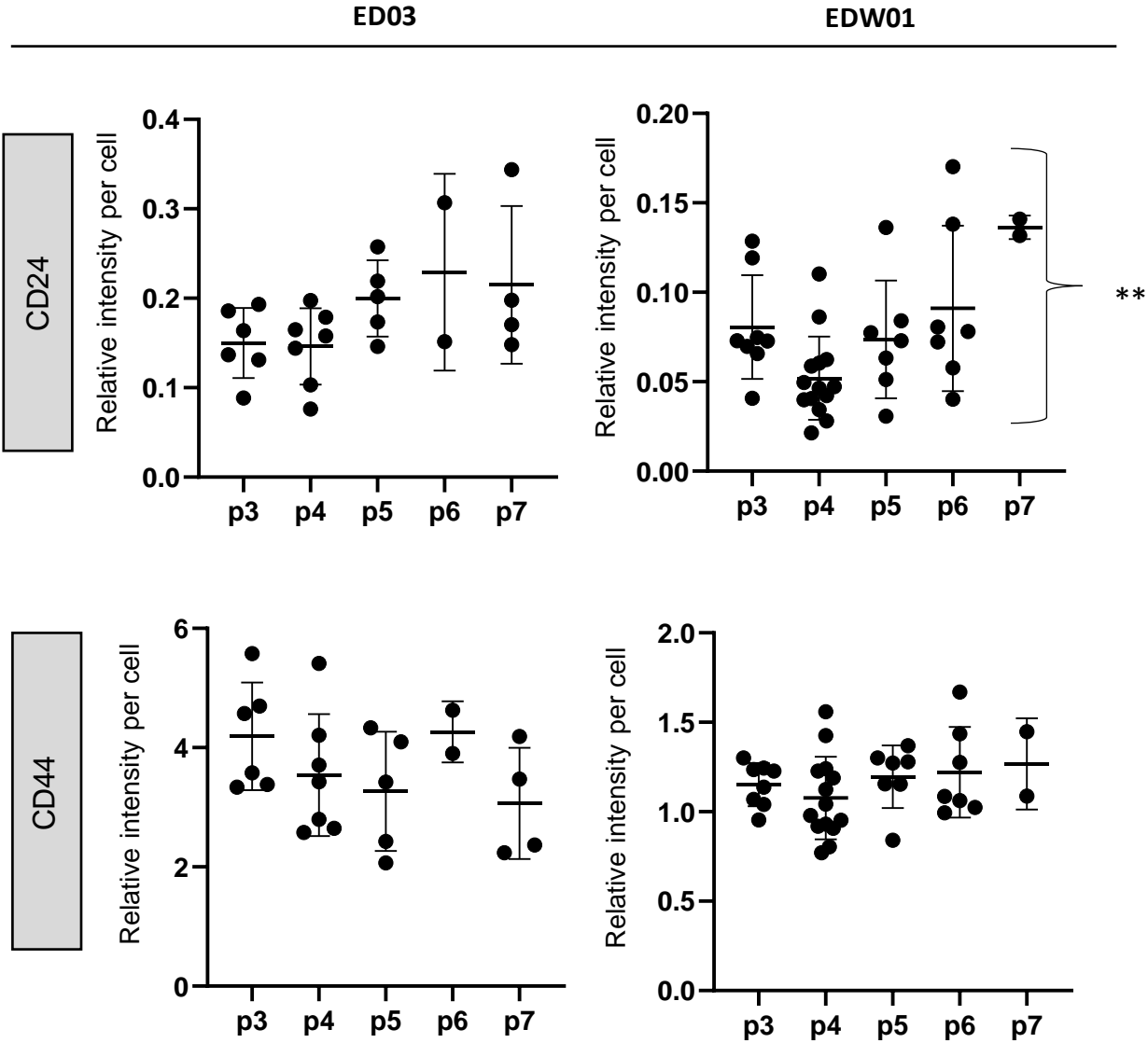

Supplement: Supplementary file 5 — Additional file 5: Supplementary Fig. 5. Image J-based quantification of relative intensity per cell of IHC targets that displayed the greatest visual change between PDXs and across passages 3 to 7 for both PDXs: CD24 and CD44. Relative intensity per cell was calculated by dividing the overall area of DAB positivity for the IHC target by overall nuclear area. Statistical significance was determined by an Ordinary one-way ANOVA, where * indicates p < 0.05 and ** indicates p < 0.005. [file 13058_2020_1366_MOESM5_ESM.pdf]

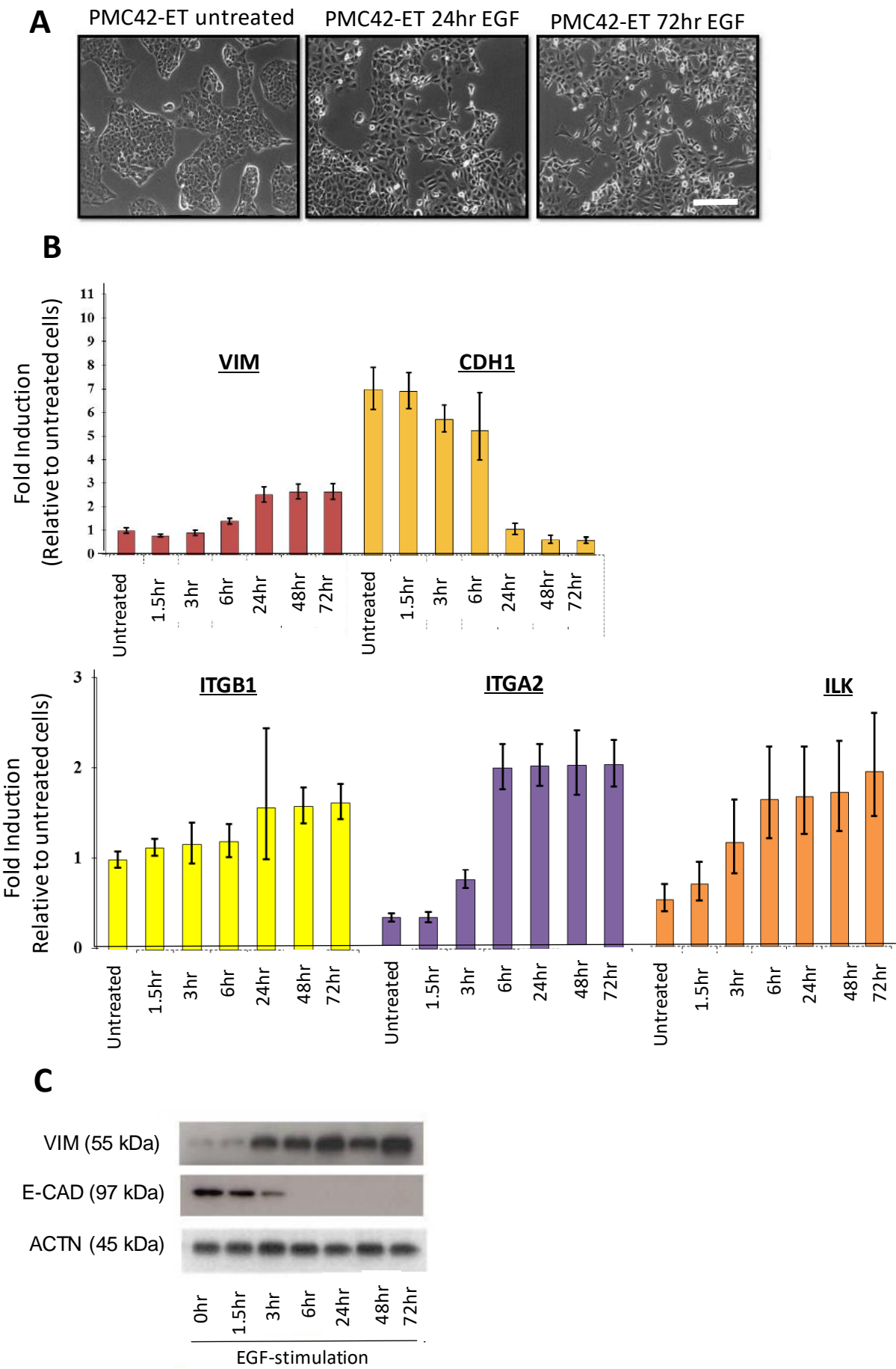

Supplement: Supplementary file 6 — Additional file 6: Supplementary Fig. 6 EGF treatment (10 ng/ml, 72 h) of PMC42-ET breast cancer cells resulted in an EMT which associated with an upregulation (trend only) of ITGB1, ITGA2 and ILK. A. Phase contrast morphology, B. gene expression changes as assessed by RT-qPCR. Results are from one experiment, representative of two independent experiments. Error bars are standard deviation of n = 3 technical replicates within 1 biological replicate (1 experiment). C. Western blotting for vimentin, E-cadherin, and actin across the EGF time course. Scale bar, 100 μm. [file 13058_2020_1366_MOESM6_ESM.pdf]
