# Supplementary material for: Integrin alpha-2 and beta-1 expression increases through multiple generations of the EDW01 patient-derived xenograft model of breast cancer—insight into their role in epithelial mesenchymal transition in vivo gained from an in vitro model system
Source: Breast Cancer Res. 2020 Dec 4;22:136. doi: 10.1186/s13058-020-01366-8 (PMC7716465; doi:10.1186/s13058-020-01366-8)

Supplementary figure 2B **Wafai et al., 2020**

Donor blocks for Figure 2A

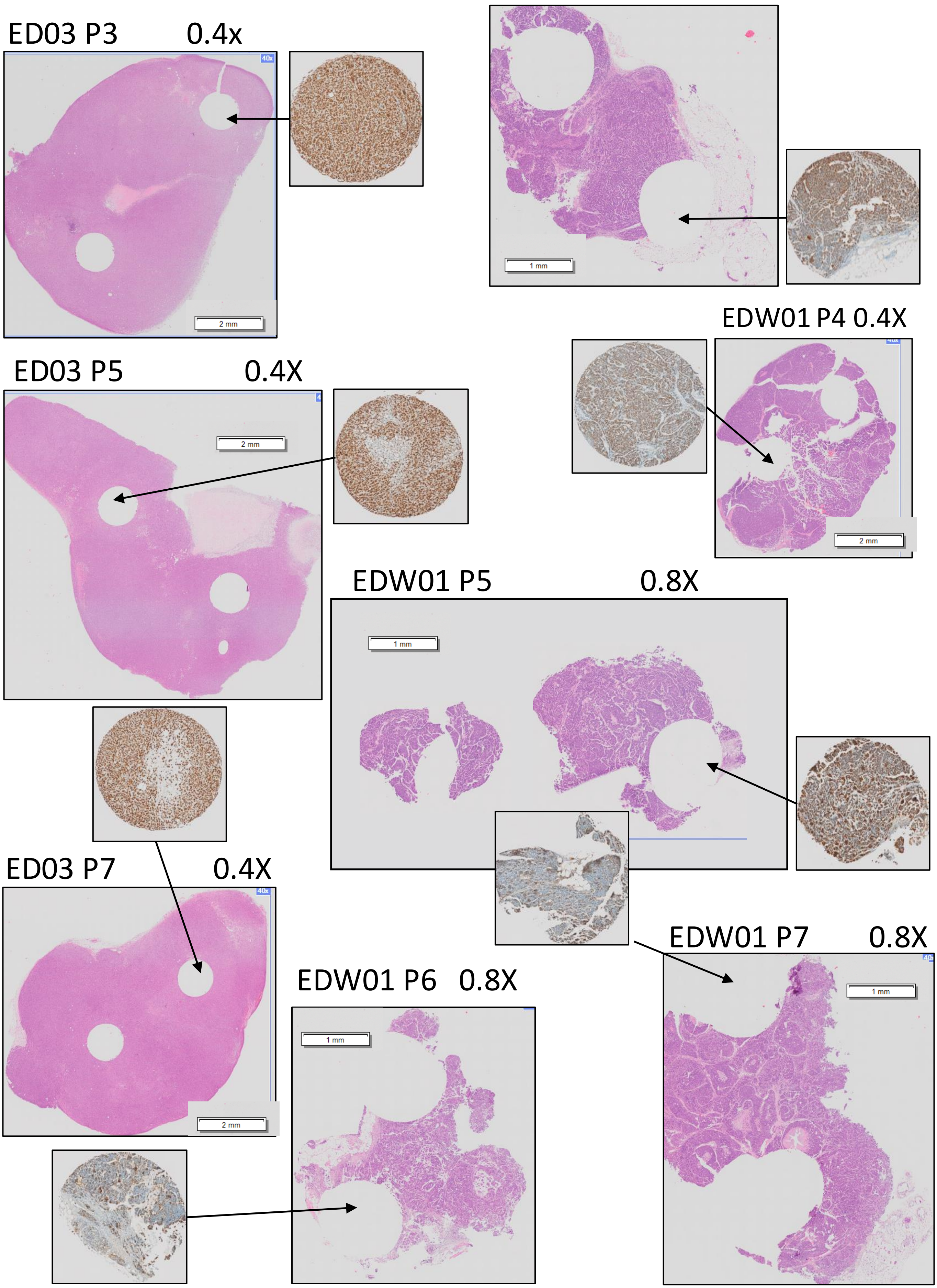

Supplement: Supplementary file 2 — Additional file 2: Supplementary Fig. 2. H&E stained sections of the donor blocks from which representative duplicate cores were taken for creation of a tissue micro-array recipient block, which was then used for all immunohistochemistry and histochemical staining depicted in this manuscript. Part A depicts donor blocks used for cores shown in Fig. 1, part B depicts donor blocks used for cores shown in Fig. 2A, and part C depicts donor blocks used for cores shown in Fig. 3. 0.4x magnification, scale bar = 2 mm. 0.8x magnification, scale bar = 1 mm. [file 13058_2020_1366_MOESM2_ESM.zip › Supplementary Figure 2B.pdf]
