# Supplementary material for: Integrin alpha-2 and beta-1 expression increases through multiple generations of the EDW01 patient-derived xenograft model of breast cancer—insight into their role in epithelial mesenchymal transition in vivo gained from an in vitro model system
Source: Breast Cancer Res. 2020 Dec 4;22:136. doi: 10.1186/s13058-020-01366-8 (PMC7716465; doi:10.1186/s13058-020-01366-8)
Supplement: Supplementary file 7 — Additional file 7: Supplementary Fig. 7. Assessment of clinical parameters (Regression Free Survival-RFS, Overall Survival-OS, Distant Metastasis Free Survival-DMFS and Progression Free Survival-PFS) in Luminal A cancers with respect to high versus low ITGB1, ITGA2 or ILK expression, as derived from a previously published database [65]. [file 13058_2020_1366_MOESM7_ESM.pdf]

Gene	Logrank P value (number of patients) – LUMINAL A only			
	**RFS**	**OS**	**DMFS**	**PPS**
ITGA2	NS (1933)	NS (611)	NS (965)	NS (179)
ITGB1	NS (841)	NS (271)	NS (281)	NS (173)
ILK	0.0000085 (1933)	NS (611)	NS (965)	NS (179)
